# Mucins in Intestinal Mucosal Defense and Inflammation: Learning From Clinical and Experimental Studies

**DOI:** 10.3389/fimmu.2020.02054

**Published:** 2020-09-04

**Authors:** Jensine A. Grondin, Yun Han Kwon, Parsa Mehraban Far, Sabah Haq, Waliul I. Khan

**Affiliations:** ^1^Farncombe Family Digestive Health Research Institute, McMaster University, Hamilton, ON, Canada; ^2^Department of Pathology and Molecular Medicine, McMaster University, Hamilton, ON, Canada

**Keywords:** mucins, goblet cell, mucosal defense, intestinal inflammation, enteric infection

## Abstract

Throughout the gastrointestinal (GI) tract, a distinct mucus layer composed of highly glycosylated proteins called mucins plays an essential role in providing lubrication for the passage of food, participating in cell signaling pathways and protecting the host epithelium from commensal microorganisms and invading pathogens, as well as toxins and other environmental irritants. These mucins can be broadly classified into either secreted gel-forming mucins, those that provide the structural backbone for the mucus barrier, or transmembrane mucins, those that form the glycocalyx layer covering the underlying epithelial cells. Goblet cells dispersed among the intestinal epithelial cells are chiefly responsible for the synthesis and secretion of mucins within the gut and are heavily influenced by interactions with the immune system. Evidence from both clinical and animal studies have indicated that several GI conditions, including inflammatory bowel disease (IBD), colorectal cancer, and numerous enteric infections are accompanied by considerable changes in mucin quality and quantity. These changes include, but are not limited to, impaired goblet cell function, synthesis dysregulation, and altered post-translational modifications. The current review aims to highlight the structural and functional features as well as the production and immunological regulation of mucins and the impact these key elements have within the context of barrier function and host defense in intestinal inflammation.

## Introduction

The mammalian gastrointestinal (GI) tract harbors a dynamic and complex ecosystem with gut microbes, food particles, foreign substances and the host cells participating in constant interaction. As such, the host requires relentless surveillance and persistent protection in order to maintain strict homeostatic conditions. As the bridge between the internal and external environments, it is no surprise then that roughly 70% of the immune system resides within the GI tract (1). Though these imperative defensive mechanisms span the initial innate responses to the more complex adaptive pathways, the physical aspects of protection should not be overlooked. One such physical aspect is the mucus layer of the GI tract which is responsible for providing lubrication for the passage of food, protecting the underlying epithelium from commensal microbes and establishing a physical barrier against invading pathogens, as well as toxins and other environmental irritants (2, 3). The mucus layer, particularly through its transmembrane components, also influences several cell signaling pathways that can modulate inflammatory responses, impact cell-cell interactions as well as regulate proliferation, differentiation and apoptosis (4–6).

**Figure 1:**
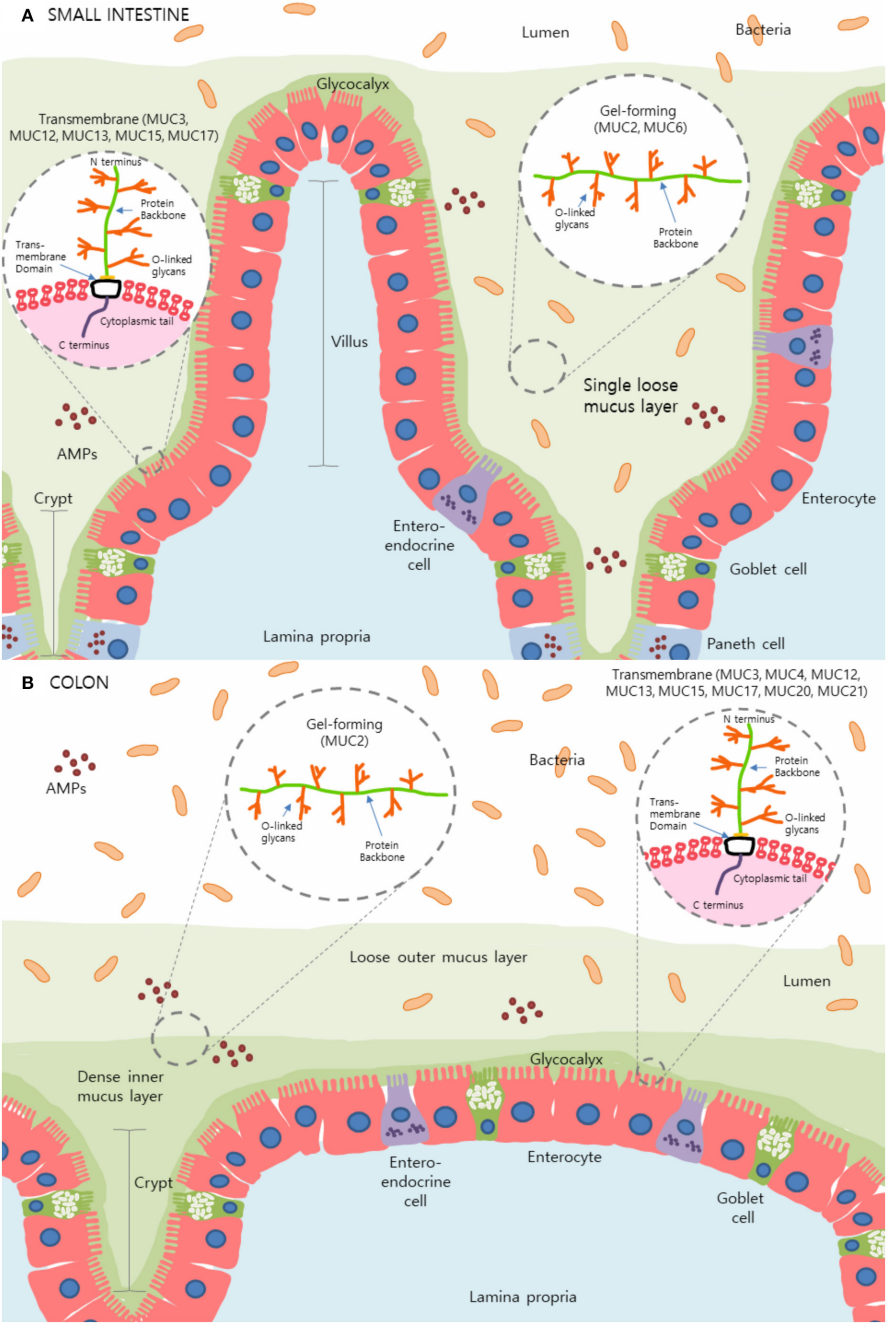
The mucus layer of the small intestine and colon. **(A)** In the small intestine, only one layer of loosely attached mucus is present and is penetrable by resident microbes. **(B)** Primarily produced by goblet cells, colonic mucus is comprised of two layers: an outer layer permeable to bacteria and a tightly adhered inner layer impermeable to bacteria. Here, secreted gel-forming mucins, largely MUC2, are the main components of this mucus layer and provide its viscoelastic properties. Transmembrane mucins including MUC3A/B, MUC12, MUC13, MUC15, and MUC17, form a carbohydrate-rich layer called glycocalyx lying between the secreted mucins and the underlying epithelial cells in both the small intestine and colon. Simplified structures of transmembrane mucins and gel-forming mucins can be seen in the magnified sections. Transmembrane mucins are generally comprised of two subunits; the heavily glycosylated and larger extracellular subunit and the shorter subunit consists of a small extracellular domain, a transmembrane domain and a cytosolic compartment. The extracellular protein backbone contains tandem repeat units of varying lengths consisting of the amino acids proline, serine, and threonine which create binding sites for O-linked oligosaccharides. This protein backbone and O-linked glycan structure are also present in secretory/gel-forming mucins.

The intestinal mucus layer is principally comprised of a subset of high molecular weight glycoproteins called mucins, which play a crucial role in physical protection as well as in regulating the concentration and passage of water, ions, and other immune mediators such as antimicrobial peptides (AMPs) and immunoglobulin-A (IgA) within the gut (2, 3, 7). Uniquely, the stomach and colon contain a dual-layer of mucus that is composed of polymeric sheets of these highly glycosylated mucins (Figure 1). These two layers can be categorized into the dense inner layer which is firmly attached to the epithelial cells below and impermeable to bacteria, vs. the outer layer which is loosely attached to, and easily removed from, the dense underlying layer (8, 9). This outer layer is also penetrable by bacteria. In contrast, the small intestine contains only one loose layer of mucus, which is penetrable by bacteria [Figure 1; (8, 9)].

Goblet cells, as well as the three other principal cells (enterocytes, enteroendocrine cells, and Paneth cells) of the gut mucosa arise from multipotent stem cells at the base of crypts of Lieberkühn (10). Among these unique cell types, goblet cells, are chiefly responsible for the production and preservation of the mucus blanket via mucin production and are heavily influenced by interactions with the immune system (3). Enterocytes also minorly contribute to the production of secreted mucins (11–13). It should also be noted that the distribution and density of goblet cells within the GI tract varies; numbers increase distally and reach a peak in the distal ileum and rectum (14). Within the stomach, mucus production is vital to protect the gastric mucosa from digestive enzymes and the harsh acidic environment of the lumen. Of the five main cell types that contribute to the biochemical milieu of the gastric lumen (including parietal cells, chief cells, and enterochromaffin-like cells), surface mucus cells or foveolar cells, and mucus neck cells are, as the names suggest, the main producers of gastric mucus (15–18). Within the distinct regions of the stomach, the organization of the invaginations that house these cells varies. In the proximal corpus, stem cells are largely located within the isthmus and are confined to the upper third of the gastric pits. In contrast, stem cells in the distal antrum are typically located in the bottom third of the invagination. In either case, these multipotent progenitor cells can move bidirectionally to either the mucosal surface or the base of the gastric pits differentiating and maturing into the principal epithelial cells of the stomach as they do so (19). Over the course of their migration, those cells destined to become surface mucus and mucus neck cells differentiate and gradually release mucin glycoproteins into the lumen (15).

Given the primary role of the mucus layer in physical defense, the influence that it has on GI inflammatory pathologies is increasingly of interest. Often these pathologies are accompanied by impaired goblet cell function as well as dysregulated mucin biosynthesis with considerable qualitative and quantitative changes (20, 21). One group of diseases that is strongly influenced by the proper function of goblet cells and their secreted mucins is inflammatory bowel disease (IBD) which is broadly classified into Crohn's disease (CD) and ulcerative colitis (UC) (22). These conditions are characterized by chronic inflammation of the GI tract and are increasing in prevalence, particularly as newly industrialized countries become progressively more “westernized” (23). Unfortunately, both cause and cure remain elusive. Colorectal cancer also presents alterations in GI mucin production and function. Lastly, bacterial and parasitic infections, which are more prevalent in developing countries, are also associated with mucin dysfunction and inflammation of the GI tract (3, 24). The present review aims to highlight the structural and functional features as well as the production and immunological regulation of mucins, and the impact these key elements have within the context of barrier function and host defense in intestinal inflammation.

## Structural Features and Classification Of Mucins

Thus far, more than 20 mucin genes have been identified (25). Though the corresponding glycoproteins associated with each of these genes have distinct differences, mucins, in general, share conserved structural features. The protein backbone contains tandem repeat units of varying length consisting of the amino acids proline, serine, and threonine, which create sites for O-glycosylation by O-linked oligosaccharides (26). The majority of these O-linked oligosaccharides are composed of N-acetyl galactosamine (GalNAc), N-acetyl glucosamine (GlcNAc), galactose (Gal), fucose (Fuc), and are often terminated by sialic acids (2, 26, 27).

The O-glycosylation process originates with GalNAc attachment to either a serine or threonine residues within the protein backbone, which can then be further elongated by additional carbohydrate residues. These initial additions can be classified as one of six common “core” regions which encompass the GalNAc-peptide attachment point and any sugars directly linked to this GalNAc. This core region is either terminated by a sialic acid residue or extended to form the backbone and, eventually, the peripheral regions of the glycan, the addition of which marks the termination of O-glycosylation (28). Variation in both the number and type of residues added, as well as, substrate availability and competition among transferases generates immense structural variability in mucin glycoproteins and results in a range from short linear structures to more complex branched forms (28). Within the mucin structure, O-linked oligosaccharides can be added as frequently as one in three amino acids and can substantially increase the molecular weight of mucins and help attract water to the mucus layer. The O-glycosylation of mucins also provides these proteins resistance to the activity of proteases and helps to prevent degradation (28, 29).

Mucins can be broadly classified into gel-forming or transmembrane based on their structural and functional features. Secreted gel-forming mucins, such as MUC2, MUC5AC, MUC5B, and MUC6, are the main components of the mucus layer and provide its viscoelastic properties (Figure 1). These mucins undergo homo-oligomerization through the formation of disulfide (S–S) bonds at their cysteine-rich N- and C-terminals aiding in the creation of a flexible network (11, 30). In the intestine, MUC2 is the predominant gel-forming mucin that contributes to the formation of the mucus barrier. MUC5AC, which is normally present in the stomach, can also be upregulated within the intestines during enteric infection (31) suggesting these mucins may have crucial roles at multiple mucosal sites. MUC5B can also be expressed in low levels in the colon while MUC6 is preferentially expressed in the stomach and duodenum (32, 33). MUC7 is a secreted mucin found in saliva and within the oral cavity and is often categorized separately from the rest of the secreted mucins (26). The differential classification of MUC7 is due to its low molecular weight and the fact that it does not significantly contribute to the viscoelastic properties of mucus (34). Unlike other gel-forming mucins, MUC7 lacks cysteine-rich terminals, does not polymerize and exists primarily as a monomeric structure (35).

Transmembrane mucins, such as MUC1, MUC3, MUC4, MUC13, and MUC17, are expressed on the apical surfaces of epithelial cells and form a carbohydrate-rich layer called glycocalyx which acts as a protective barrier between the secreted mucins and the underlying epithelial cells [Figure 1; (36, 37)]. The rigid structure of these mucins spans the length of the cell membrane and participates in intracellular signaling with their C-termini located inside the cell (11, 38). Transmembrane mucins are generally comprised of two subunits held together by non-covalent sodium dodecyl sulfate-labile bonds. The larger subunit, which is primarily extracellular, contains serine and threonine repeat units and is heavily glycosylated. The shorter subunit is comprised of a small extracellular domain, a transmembrane domain, and a cytosolic compartment (39). Although the exact mechanisms remain unknown, transmembrane mucins have been heavily implicated in cell signaling (6). The function MUC1 displays in the downregulation of the Toll-like receptor (TLR)-initiated innate immune response is a well-established instance of cell signaling by transmembrane mucins (40).

## Goblet Cell Differentiation, Mucin Production, and Secretion

The major specialized intestinal epithelial subtypes including goblet cells, Paneth cells, and enteroendocrine cells, originate from stem cells located at the base of intestinal crypts and are derived from a common secretory precursor cell. Enterocytes, the most abundant cell type within the epithelial layer of the gut, develop from non-secretory lineages of stem cells (41).

Lineage differentiation of these specialized intestinal epithelial cells is, in part, regulated by Notch signaling. Notch is a cell-surface receptor which is responsible for the regulation of various DNA-binding proteins (42). Although the exact mechanism is not known, inhibition of the Notch pathway leads to preferential differentiation of intestinal stem cells into goblet cells, the primary producers of secreted mucins within the GI tract (14, 43, 44).

During their maturation, goblet cells undergo substantial morphological changes as they migrate from the base of intestinal crypts to the villi. At the base of the crypt, stem cells are fated into early goblet cell lineages via Wnt-signaling, which give rise to immature goblet cells. These immature cells are large in size, pyramidal, and contain mucin granules interspersed among the organelles. During maturation, goblet cells begin to lose cytoplasmic volume (14, 45). In this process, SAM pointed domain-containing ETS Transcription Factor (SPDEF) drives terminal differentiation into mature goblet cells (46). The apical region of mature goblet cells, called the theca, is cup-like in appearance and is packed with mucin granules, while the organelles and nucleus congregate to the basal stem of the cell (14, 47). In addition to morphological changes, goblet cell differentiation is also accompanied by alterations in the chemical composition of the produced mucins. During maturation, secreted mucins become more acidic and develop more sites for N- and O-glycosylation (14, 48).

After synthesis, mucin-packed granules are transported to the apical cell surface via secretory vesicles and are released into the lumen by the processes of basal secretion or compound exocytosis/regulated secretion (49). Basal secretion, occurring under normal physiological conditions, involves the continuous fusion and release of single mucin granules into the gut lumen. This steady and unstimulated release maintains the thickness of the mucus barrier and protects the underlying epithelia from the constant threat of luminal contents (50). Compound exocytosis, on the other hand, is a process induced or stimulated by inciting factors such as microbial products, hormones, inflammatory cytokines and neurotransmitters such as acetylcholine (26, 49, 51–53). Here, centrally stored mucin vesicles rapidly fuse and empty their contents in response to these secretagogues (54). Since the 1980s, the morphological process, molecular mechanism, and relationship between immune and non-immune regulators of compound exocytosis have been active areas of research.

## Immunological Regulation of Mucin Production

The immune response can be broadly divided into the innate and adaptive systems. The innate system relies on evolutionarily conserved, somatically-encoded receptors to recognize molecular patterns on microbes (55, 56). In contrast, the adaptive immune response relies on the specific recognition of pathogenic antigens for initiation of said immune response (57).

### Innate Immunological Regulation

Within the innate domain, pattern recognition receptors (PRRs) such as TLRs and cytoplasmic nucleotide-binding oligomerization domain (NOD)-like receptors (NLRs) play an essential role in mucin synthesis (58). TLRs are a family of 11 evolutionarily conserved transmembrane receptors which are located on the cell surface or on intracellular endosomes. These receptors are activated by pathogen-associated molecular patterns (PAMPs). This activation terminates with the induction of the NF-κB family of transcription factors and the upregulation of the immune response (25, 59).

Expression profiles of TLRs differ throughout the GI tract. Recently, by utilizing five strains of TLR reporter mice, the spatial expression of TLRs in intestinal epithelial tissue was visualized. TLR2, 4, 5, 7, and 9 were found to be minimally or not expressed in the small intestinal epithelium while TLR2, 4, and 5 expression levels were substantially higher in the colon (60). Further, TLR3 was expressed at similar levels in both small intestinal and colonic epithelial cells (60). Only TLR5 was expressed by goblet cells in the small intestine while colonic goblet cells were found to express TLR1, 2, 4, and 5 (61) suggesting intrinsic TLR-meditated mucin regulation in these cells.

Through their activation of TLRs, ligands such as lipopolysaccharide (LPS) found on the outer membrane of most Gram-negative bacteria, lipoteichoic acid (LTA) on the cell wall of Gram-positive bacteria, and flagellin found in bacterial flagella are all potent activators of MUC2 expression (25, 62). For instance, upon stimulation by their respective ligands TLR2/1, TLR4, and TLR5, promote the downstream activation of the NLRP6 inflammasome in subpopulations of sentinel goblet cells located at the entrance of colonic crypts. This inflammasome, a multiprotein complex located in the cytoplasm of these cells, functions as a sensor for cellular stresses and plays a key role in intestinal barrier maintenance, infection defense and mucosal renewal (54, 63). These TLR-initiated cascades stimulate the compound exocytosis of MUC2 and trigger mucin secretion from adjacent goblet cells through intercellular gap junction signals. This subsequent increased MUC2 secretion can thus aid in the expulsion of bacteria from the upper part of the crypts (54). Recent studies have shown that TLR4 activation is important in goblet cell response during *Citrobacter rodentium* infection (64) and can regulate the differentiation of goblet cells in intestinal organoids (65). The activation of TLR4, involving the binding of lipid A moiety of LPS to the LPS binding protein (LBP), can upregulate the expression of MUC2 through the Ras-MEK1/2-Erk1/2 and NF-κB pathways (66). Establishing whether the particular crypt location of the goblet cells is a determining factor in mucin production in response to various TLR ligands will be a worthwhile direction for future research.

Further evidence gleaned from genetic knockout models have helped highlight the differential effects of TLRs on mucin regulation. For instance, naïve *Tlr1*^−/−^ mice have defective production and/or secretion of MUC2 in the colon leading to a patchy and significantly depleted mucus layer (67). *Tlr5*^−/^^−^ mice display a mosaic phenotype; a subset of these mice develop spontaneous colitis while the majority do not. Interestingly, compared to wild-type mice, colitic *Tlr5*^−/^^−^ mice do not display the normal dual-layer of colonic mucus; only a disorganized and largely penetrable layer is present. In contrast, non-colitic *Tlr5*^−/^^−^ mice had a normal though slightly thinner mucus layer (68).

Scant research exists involving the interaction between TLRs, goblet cell function, and mucin regulation in intestinal parasitic infection *in vivo* and this area remains largely unexplored. *In vitro*, however, antigens from the intestinal trematode *Gymnophalloides seoi* have been found to induce the expression of both TLR2 and MUC2 in HT-29 cells in an IFNγ-dependent manner. Further, co-stimulation with *G. seoi* antigen and antibodies against both TLR2 and TLR4 have been shown to diminish MUC2 expression in HT-29 cells compared to those cells treated with the antigen only. Thus, the authors of this study hypothesize that the induction of MUC2 expression as an antiparasitic response in human IECs, may, at least in part, be a result of TLR activation (69). Additional *in vivo* and *in vitro* research will provide valuable insights into the interaction between TLRs, goblet cell function and mucin regulation in parasitic infection.

In contrast to the transmembrane TLRs, NLRs are a family of innate intracellular receptors (70). However, similar to TLR signaling, activation of NLRs such as NOD1 and NOD2 by intracellular ligands (i.e., bacterial peptidoglycans) ultimately results in the activation of important transcription factors, such as NF-κB, to induce immune responses (71). An enteric infection model using the helminth, *Trichuris muris*, has revealed that NOD1 and NOD2 receptors are necessary for MUC2 synthesis and parasitic expulsion; knocking out both of these genes (Nod-DKO) result in infected mice with lower goblet cell numbers and decreased MUC2 expression (72).

Other receptors can also impact mucin production via the innate response. LTA from Gram-positive bacteria can upregulate MUC2 expression by acting on platelet-activating factor receptor (PAFR) and, through a multistep process, activate the Ras-MEK1/2-Erk1/2 and NF-κB pathways (25). Moreover, flagellin signals through the glycolipid receptor asialoGM1 (ASGM1) ultimately lead, again, to upregulated expression of MUC2 (73). ASGM1-mediated upregulation of MUC2 involves the sequential activation of phospholipase C, an increase in calcium ion levels as well as ERK1/2 and NF-κB activation (73).

### Dendritic Cells and Macrophages

In the local draining lymph node, antigen presenting cells (APCs) such as dendritic cells and macrophages present the phagocytosed and processed antigen to aid in the differentiation of naïve CD4^+^ T cells to Th2 cells. These cells then secrete effector cytokines such as IL-13 and, thusly, contribute to mucus production and goblet cell hyperplasia (74). In addition, macrophages, primed by the canonical type 2 cytokines IL-4 and IL-13, can transition into alternatively activated or M2 macrophages (75). Moreover, alternatively activated macrophages can be generated by IL-33 and polarization was associated with increased induction of IL-13 (76), suggesting these APCs may also play a more direct role in the regulation of mucin via this cytokine.

### Innate Lymphoid Cells (ILCs)

ILCs are a recently discovered group of innate immune cells that play an essential role in host immunity, tissue protection, and adaptive immune regulation, particularly within the intestinal mucosal barrier (77–79). ILCs bridge the gap between the innate and adaptive immune responses by producing immune-regulatory cytokines. Based on the effector cytokines that ILCs secrete, the transcription factors that regulate their development, and markers dotting their cell surfaces, this family of cells can be subdivided into three groups: ILC1, ILC2, and ILC3 (77, 78).

#### ILC1

T-bet, a transcription factor expressed by ILC1s, is widely known as an important regulator for type 1 immunity. However, it has also been shown to protect against intracellular pathogens, such as *Salmonella enterica*. During *Salmonella* infection, ILC1s play an important role by producing IFN-γ and, thus, driving the secretion of mucus-forming glycoproteins (80).

#### ILC2

ILCs bridge the gap between the innate and adaptive immune responses by producing immune-regulatory cytokines. It is becoming increasingly apparent that ILCs, particularly ILC2, have emerged as a crucial innate immune cell critical for the production of mucin through T helper 2 (Th2) immune responses. ILC2s arise from common lymphoid progenitor (CLP) cells (81) and express the transcription factors, retinoic acid receptor-related orphan receptor α (RORα) and GATA binding protein 3 (GATA3) (82). Mature ILC2s respond to epithelial cell-derived cytokines including IL-25, IL-33, and thymic stromal lymphopoietin (TSLP) to produce Th2 cytokines such as IL-4, IL-5, IL-9, and IL-13 (83–86). These effector cytokines support the development of type 2 inflammation as well as mucin production in the context of parasitic immunity and allergic diseases (82). Recently, the function of these cells in helminth infection resistance has been demonstrated, particularly with regards to the impact of IL-13-secreting ILC2s on mucin-producing goblet cells. IL-33 has been shown to indirectly induce intestinal goblet cell differentiation and MUC2 expression via IL-13-secreting ILC2s (87). Moreover, IL-33-deficient (*Il33*^−/−^) mice fail to expel *Nippostrongylus brasiliensis* worms due to impairment of ILC2 (88), further demonstrating the essential role of ILC2s in helminth infection immunity.

#### ILC3

ILC3s are also implicated in the maintenance of gut homeostasis. ILC3s express the transcription factor, RORγt, and IL-22, one of the effector cytokines secreted by ILC3s (89). Upon binding to its receptors, IL-22R1 and IL-10R2, on the intestinal epithelial cells, IL-22 induces mucin generation and goblet cell hyperplasia (90, 91). In addition, IL-22 promotes the activation of NOD signaling which leads to mucin secretion by goblet cells (92).

### Adaptive Immunological Regulation

Unlike the innate immune system which relies on germ-line encoded PRRs, the adaptive immune system generates specific receptors to recognize the substantial diversity of harmful antigens through a process called somatic recombination (93). The principal cell types of the adaptive immune system are T and B lymphocytes which are vital in maintaining gut homeostasis as well as host protection in GI diseases (94). Consequently, T lymphocytes play an important role in the regulation of mucin release by goblet cells (95). Initial studies demonstrated that during *N. brasiliensis* infection, anti-CD4 antibody treatment in mice prevented spontaneous recovery. These mice also displayed T-helper cell depletion along with a reduction in mucin levels, despite unchanged goblet cell counts (96).

The immune response to intestinal helminth infection is characterized as a Th2-dominant response accompanied by the upregulation of cytokines, such as interleukin (IL)-4, IL-5, and IL-13 (97, 98). Animal models of enteric infections including *N. brasiliensis* (99), *Strongyloides ratti* (100), *T. muris* (98), and *Trichinella spiralis* (101) have shown that these helminth infections are also accompanied by goblet cell hyperplasia and an increase in mucin production and secretion that aid in worm expulsion (102). It is postulated that these changes in mucin production and goblet cell hyperplasia are at least partially the result of the Th2-mediated immune response (103). As an activator of Th2 immune responses, signal transducer and activator of transcription factor 6 (Stat6) has been identified as a critical inducer of goblet cell hyperplasia (3, 104). This factor's crucial role is neatly illustrated in *Hymenolepis diminuta* infection; STAT-6 knockout mice infected with this tapeworm were unable to clear this infection due, in part, to diminished goblet cell response, unlike their IL-13 and IL-4 deficient counterparts at 12 days post-infection (105). Interestingly, it has been shown that goblet cell hyperplasia may also occur in a Th2-independent manner without the influence of IL-4 and IL-13 in some parasitic infections, including *Schistosoma mansoni* (106). It should also be noted that with the discovery of ILC2s, our understanding of the type 2 immune response has expanded. Both ILC2 and Th2 cells can be activated by IL-33, IL-25, and TSLP, and release type 2 effector cytokines (e.g., IL-5 and IL-13) during parasite infections, and thus, contribute to type 2 immunopathology. Further, Th2 cells respond to antigen stimulation by dendritic cells which also receive signals from cytokines released by ILC2s (107). These findings suggest that Th2 immune responses may be initiated by ILC2 since the activation of ILC2s occurs during the early phase of type 2 immune responses (108, 109).

Moreover, emerging evidence suggests a novel role for other cytokines in mucus production. Previously considered a non-factor in mediating parasitic expulsion, IL-22, a member of the IL-10 family of cytokines, has been shown to induce goblet cell hyperplasia and mucin release. IL-22-deficient (*Il22*^−/−^) mice have defective goblet cell responses during *N. brasiliensis* infection despite strong Th2 cytokine induction (110). Furthermore, evidence suggests that, following infection with *T. trichiura*, the human gut accumulates IL-22-producing Th cells within the intestinal mucosa and the resultant increase in IL-22 production and Th2 cytokines promotes goblet cell hyperplasia and mucus production (111). Similarly, humans infected with *Necator americanus*, a parasitic hookworm, showed upregulated IL-22 production as well as a robust systemic and mucosal Th2 and T-regulatory (T_reg_) response, ultimately promoting goblet cell hyperplasia and worm expulsion (112). In addition to quantitative changes, cytokines can also regulate the quality and composition of mucins. A recent study found that administration of IL-10 reduces endoplasmic reticulum (ER) stress and prevents the misfolding of MUC2 in an *in vitro* model (LS174T) of intestinal goblet cells (113). Consequently, T lymphocytes play an essential role in controlling the release of mucins through signaling by Th2 cytokines (e.g., IL-4, IL-5, and IL-13) and other mediators such as IL-22.

In addition to Th2 cytokines, several Th1 cytokines have been shown to regulate mucin biosynthesis. In the intestinal cancer cell line, LS180, it was observed that the pro-inflammatory cytokines, including IL-1, IL-6, and TNF-α, increased the expression of MUC2 mRNA (95). Due to the influx of Th1 cytokines, reduced mucin glycosylation via incomplete processing of N-glycans was also present (95). In addition, several *in vitro* and *in vivo* models have demonstrated the relationship between downregulated MUC2 expression and increased IL-6 in the context of colon cancer. Interestingly, this inverse relationship tends to be associated with liver metastasis and the promotion of tumor growth in mice (114, 115). Further, in BALB/c duodenal explants, macrophage-derived IL-1 and human rIL-1β have the ability to induce mucin secretion from goblet cells (116). The authors suggest that this dose-dependent process may act as a protective mechanism by aiding in the clearance of toxic substances from the gut in periods of mucosal inflammation (116). In contrast, in a murine model of *C. rodentium* infection, the Th1 cytokines, IFN-γ, and TNF-α, decreased intestinal mucin production and its speed of transport from the Golgi to secretory vesicles. Lending further support to this finding, *in vitro* treatment of infected and non-infected intestinal mucosal surfaces with IFN-γ and TNF-α decreased the number of goblet cells, mucus thickness and transport (117). The contrasting data in the above paragraph implies that the effect of Th1 cytokines on mucin synthesis, transport, and secretion depends critically on, not only, the type of cytokine but also the pathological process in question.

**Figure 2:**
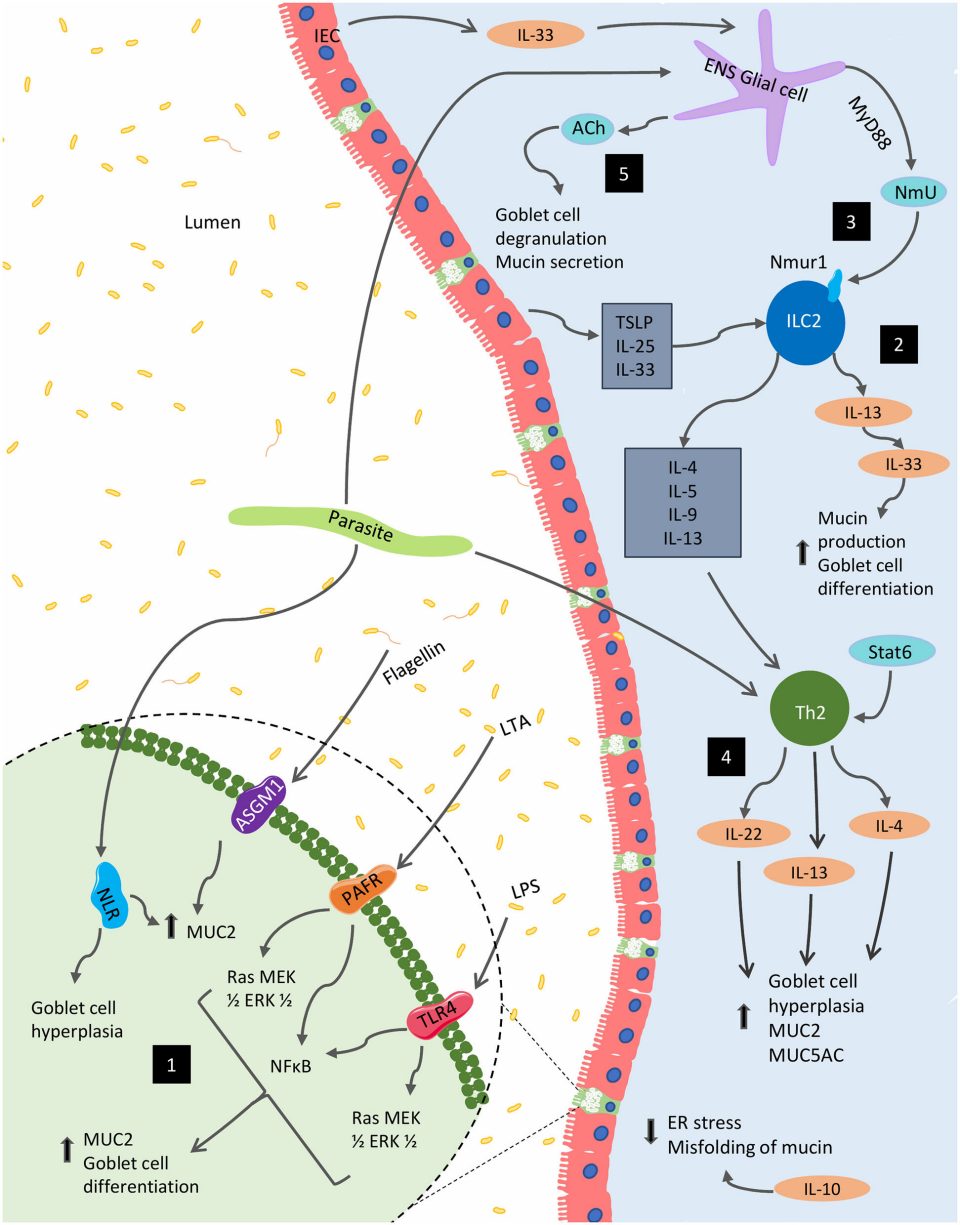
Immunological regulation of mucin production and goblet function within the GI tract. Several immunological factors regulate and alter the production of mucins and the goblet cell function within the gut. (1) Bacterial components, including lipopolysaccharide (LPS), lipoteichoic acids (LTA) and flagellin, are potent activators of MUC2 expression via Toll-like receptors (TLRs). The latter two of these components can also stimulate MUC2 expression via platelet-activating factor receptor (PAFR) and glycolipid receptor, asialoGM1 (ASGM1), respectively. In parasitic models, NOD-like receptors (NLRs) are necessary for MUC2 synthesis and parasitic expulsion. (2) In the context of parasitic infection, mature ILC2s respond to epithelial cell-derived cytokines including IL-25, IL-33, and TSLP to produce IL-4, IL-5, IL-9, and IL-13; supporting the development of type 2 inflammation, as well as, mucin production. (3) These cells are also impacted by NmU derived from glial cells stimulated by the ESPs of certain parasites as well as IEC-derived IL-33. (4) Enteric parasitic models also incite Th2 cytokines, IL-4, and IL-13 promote goblet cell hyperplasia and mucus production. (5) From the enteric nervous system (ENS), acetylcholine (ACh) plays a role in for goblet cell degranulation and can induce mucin secretion. Several Th1 cytokines such as IL-1β, IFN-γ, and TNF-α can also regulate mucin biosynthesis (not shown).

Though extensive, the presented results highlight the need for further exploration of mucin's diverse immunological functions and the impact that the immune system itself has on mucin production. Indeed, these findings establish the crucial role mucin plays in barrier and immune function as well as the importance of immune-associated signaling on the regulation of mucin composition and function. Figure 2 highlights various immunological regulations that affect mucin production and goblet cell function within the gut.

### Enteric Nervous System Regulation

It is increasingly apparent that the enteric nervous system (ENS) also contributes to mucin production. Recent studies have shown that mucosal neurons, which are situated in close proximity to lamina propria immune cells (e.g., APCs and ILCs), interact with epithelial cells and act as key regulators of mucin production at intestinal mucosal surfaces. Acetylcholine (ACh) is a primary parasympathetic neurotransmitter that is released by preganglionic nerve fibers and the vagus nerve (118). Previously, it has been established that ACh is important for goblet cell degranulation and can induce mucin secretion (54, 119, 120).

Muscarinic ACh receptors (MRs) are one of the two types of cholinergic receptors along with nicotinic ACh receptors (NRs) (121). Of increasing interest, type 3 muscarinic receptors (M3Rs) have received significant attention for their key role in maintaining mucosal barrier function (122) and in regulating mucin production and secretion within the GI tract (123). In addition, M3Rs contribute to host defense against *N. brasiliensis* (124, 125) and *C. rodentium* (121). Despite *N. brasiliensis* infection, mice deficient in the muscarinic acetylcholine receptor M3 (*Chrm3*^−/−^) show an absence of typical goblet cell expansion (125). These mice also display impaired immunity to *C. rodentium* along with decreased goblet cell number and MUC2 gene expression in the colon compared with WT mice on day 13 post-infection (121).

Similar to MRs, vasoactive intestinal peptide (VIP) also participates in regulating intestinal goblet cell numbers and function at baseline; VIP-deficient mice show impaired goblet cell development and reduced expression of MUC2 (126, 127). VIP is a 28-amino-acid peptide secreted by enteric neurons and regulates gut motility (126). There are three known receptors for VIP: the higher affinity receptors, VIP receptor 1 (VPAC1) and VPAC2, and the lower affinity receptor PAC1 (128). Among these, VPAC1 mRNA is the most highly expressed on the apical membrane of the intestinal epithelium and receives signals from VIP fibers (129). Although VPACs are expressed on airways (130) and ocular mucosa (131), there is no direct evidence that intestinal goblet cells express VIP receptors. However, a recent study has reported that *ex vivo* treatment with VPAC antagonists resulted in a substantial decrease in goblet cell counts in the mouse ileum indicating potential ongoing VIP regulation of goblet cell production (126). Furthermore, lamina propria ILC2 function is also regulated by VIP via VPAC2 during parasite infection (132).

Interestingly, recent studies have also indicated indirect regulation of mucin production by a neuropeptide called neuromedin U (NmU) via ILC2 (89, 133, 134). During *N. brasiliensis* infection, glial cells sense intestinal epithelial cell-derived IL-33 and *N. brasiliensis* excretory/secretory products (ESPs) and trigger NmU production in a MyD88-dependent pathway (133). NmU not only induces smooth muscle contractions but also binds to NmU receptor 1 (Nmur1) on ILC2s promoting the secretion of IL-13, and, potentially impacting mucin secretion (135).

## Mucin Production in Intestinal Inflammation: Evidence From Clinical Studies

### Inflammatory Bowel Disease

Inflammatory bowel disease (IBD) is an umbrella term which includes chronic inflammatory conditions of the gastrointestinal tract such as ulcerative colitis (UC) and Crohn's disease (CD). UC is characterized by superficial inflammation radiating proximally from the rectum, whereas CD is characterized by zones of deeply inflamed and non-inflamed tissue that can extend throughout the GI tract (136). These conditions affect millions worldwide, and the incidence is increasing globally (137).

Early studies suggested that single nucleotide polymorphisms in certain mucin genes including MUC3A, MUC3B, MUC12, and MUC17, predisposed individuals to CD and UC, however, these findings have not held up in more recent genome-wide association studies (GWAS) (138). Meta-analyses of several GWAS have more recently identified the gene encoding MUC1 (139) and the locus of leucine-rich repeat kinase 2 (LRRK2) which contains the MUC19 gene, (140) to have significant associations with CD.

Both CD and UC are accompanied by dysregulation of mucin synthesis and altered post-translational modification leading to barrier dysfunction (138). In UC, changes including reduced glycosylation and sulphation (141) as well as increased sialylation alter the efficacy of the mucins present and hinder their ability to maintain effective intestinal barrier function (20) particularly with regards to bacterial penetration (142, 143). Healthy colonic mucus is often heavily sulphated with increasing levels of sulphation extending from proximal to distal regions and conferring increasing resistance to bacterial enzymatic degradation. In both CD and UC, however, this phenomena is muted (20, 141). In inflamed tissue, goblet cell depletion is present in both UC and CD compared with controls (144) and altered mucus layer thickness has also been found in both conditions (145).

UC is associated with a relatively thin and discontinuous mucus layer, goblet cell depletion and reduced MUC2 synthesis (145–147). Reduced sulfate content of MUC2 has also been noted, though compensatory and preferential secretion of this mucin in active disease results in overall unaltered sulfate levels in the colon of UC patients (147). Interestingly, MUC5AC, a mucin not normally present in the colon, has been found in UC patients undergoing surgery (148). In addition, site-specific increases in MUC1 expression and decreases in MUC2 expression were observed in UC patients at the site of crypt abscesses and adjacent to ulceration, respectively (149). Reduction in MUC9 (150) and MUC20 (151) gene expression was also noted in both active and quiescent UC compared with healthy controls. Increased gene expression of MUC16 has also been described in UC patients with active disease as well as those in remission compared to healthy controls (151). Sialylation also plays a similar role to sulphation, increasing the resistance to enzymatic degradation. In rectal biopsies of UC patients an increased average extent of sialylation per mucin oligosaccharide was noted rather than a simple increase in oligosaccharide chains (152). Intriguingly, glycan “profiles” have been established whereby healthy controls and those UC patients with inactive disease displayed similar glycan/glycosylation patterns; abundant levels of complex and larger glycans and relatively small amounts of shorter glycans. In opposition, those with active UC displayed increased presence of shorter glycans and a marked decrease in several complex glycans (153). Furthermore, these aberrant glycosylation profiles were associated with the degree of inflammation and severity of disease (153). Similarly, in a large-scale proteomics study, van der Post et al. established a core colonic mucus proteome, a set of 29 core secreted and transmembrane proteins that form the mucus barrier in healthy controls and UC patients in remission. Several of these proteins were found to be reduced in active UC patients including core structural components, MUC2 and IgGFc-binding protein (FCGBP), and other goblet cell products including calcium-activated chloride channel regulator 1 (CLCA1) and zymogen granule protein 16 (ZG16). Interestingly, this trend occurred independent of local inflammation and was associated with increased bacteria penetrability and activation of IL-18 (143). In active UC, contributing to the thinner and penetrable mucus layer, insufficient replenishment or exhaustion of sentinel goblet cells in response to successive microbial challenges has been noted and may precede the activation of disease and/or local inflammation (142, 143).

While the relationship between UC and mucus thickness is relatively well-established, the relationship regarding CD and mucus thickness remain under dispute. Early findings suggested CD patients displayed increased mucus layer thickness compared to healthy controls (146), however, more recent work has indicated that mucus layer thickness in CD patients was not significantly different compared with healthy controls (145). In seeming contradiction, a recent systematic review and meta-analysis concluded that, on average, patients with CD have a 34% reduction in total mucin levels due to significantly decreased levels of MUC5AC, MUC5B, and MUC7 (154). Similar to UC, ileal CD has also been linked with aberrant protein expression of MUC5AC and decreased expression of MUC2 (155). Aberrant expression of MUC6 has also been noted in ileal CD (156). Moreover, in the ileum of CD patients, a marked decrease in MUC1 mRNA was also observed in inflamed vs. non-inflamed tissue from the same patient (156). Interestingly, reduced gene expression of the transmembrane mucins, MUC3 and MUC4, as well as the secreted mucin MUC5B have been noted in non-inflamed tissue sections of CD patients compared with healthy controls (156) suggesting local inflammation is not necessarily a precursor for these alterations. Alterations in oligosaccharide length have also been noted in CD patients and although it has been established that levels of sulphation remain unchanged compared with healthy controls, changes in glycosylation levels in CD patients have not been thoroughly explored (20, 141, 147, 152).

From the above evidence, it is clear that alterations in mucin expression and function play a unique role in IBD. However, because of significant dispute in the literature, it is challenging to characterize distinct mucin expression profiles associated with IBD, and it should be noted that significant heterogeneity exists.

### Colorectal Cancer

Colorectal cancer is the third most common cancer in the world and accounts for substantial mortality every year (157). The expression of secreted and transmembrane mucins is altered in colorectal cancer patients. MUC1 expression was found to be increased in colon cancer patients and was correlated with poor prognosis and metastasis (158). Though MUC1 is usually undetectable with tandem repeat peptide antibodies in healthy colons due to heavy glycosylation, this post-translational modification is reduced in colorectal cancer patients which may account for the observed differences (159, 160). In addition, MUC2 expression was found to be decreased in colorectal cancer instances, other than in mucinous adenocarcinomas (12, 161). Moreover, MUC5AC, a normal component of gastric mucus which is usually absent from the colon, was shown to be expressed *de novo* in colorectal cancer (162, 163).

## Mucin Production in Intestinal Inflammation: Evidence From Animal Experiments

### Chemical Colitis

Since the 1990s, the dextran sulfate sodium (DSS) model has been used extensively in rodents to understand the pathophysiology of colitis (164). The cytokine profile associated with this chemical model is predominantly within the Th1 immune response with the upregulation of the cytokines, IL-12, IFN-γ, and TNF-α (165). Animal studies using DSS have highlighted the important and diverse roles of mucins in modulating intestinal inflammation.

As previously mentioned, MUC2 is a secreted gel-forming mucin and the main structural component of intestinal mucus which contributes significantly to host protection in the context of intestinal inflammation. *In vivo* results show that rats placed on DSS regimen undergo goblet cell depletion; however, depending on the observed location within the colon, the expression of MUC2 was unaltered or increased (166). Under a different DSS regimen, mucin expression was shown to vary with the progression of colitis. Compared with controls, DSS-treated rats showed initial upregulation of MUC2 and MUC3, followed by a rapid reduction in expression over time (167). Within 12 h of DSS administration, decreased mucus thickness and increased mucus permeability in the colon allows commensal microbes to penetrate the inner mucus layer and reach the intestinal epithelial cells. It is thought that this early bacterial invasion plays a critical role in eliciting the infiltration of immune cells and driving the development of colitis (168).

Despite the lack of consistent expression patterns in the above results, it is evident that MUC2 is an important component of the protective mucus layer of the intestine. In fact, MUC2-deficient (*Muc2*^−/−^) mice develop spontaneous colonic inflammation characterized by weight loss, changes in stool consistency, and increased expression of pro-inflammatory cytokines (169). *Muc2*^−/−^ mice also show increased severity of inflammation and disease activity when exposed to DSS (169). Therefore, it is clear that MUC2 offers significant protection against colonic inflammation and that a well-maintained colonic mucus layer is vital in preventing murine colitis. In contrast to this distinct protective role of MUC2, not all mucins have shown to have protective functions in a DSS-induced colitis model.

Unlike MUC2, MUC4 is a transmembrane mucin and a component of the glycocalyx found in the intestine. Mice deficient in MUC4 show resistance to DSS colitis and have reduced levels of pro-inflammatory cytokines compared to wild-type animals (170). Although the mechanism behind the protective role of MUC4 deletion is unknown, it is possible that *Muc4*^−/−^ mice respond by upregulating protective mucins in a compensatory manner to resist DSS-induced colitis. In fact, *Muc4*^−/−^ mice challenged with DSS colitis have been found to have higher expression of both MUC2 and MUC3 compared to wild-type mice (170). Similarly, mice deficient in MUC1, another transmembrane mucin, develop less severe colitis accompanied by a reduction in T cell infiltration (171). The lower severity of colitis in *Muc1*^−/−^ mice may also be attributed to compensatory increases in the expression of MUC2 and MUC3 (171, 172).

### Genetic Models

Besides chemical models, genetically modified animals are also commonly utilized to study intestinal inflammation and mucin production. One such model is the IL-10 knockout murine model (173). If kept in non-germ-free housing, mice deficient in IL-10 spontaneously develop colitis. This unprompted inflammation is accompanied by a reduction in the number of mucin-producing goblet cells in the intestine (173). In contrast, mice lacking the *IL-1rn* gene, coding for the IL-1 receptor antagonist, spontaneously developed IBD-like abnormalities including increased immune cell infiltration and secretion of pro-inflammatory cytokines, and had an increased number of goblet cells in the jejunum and ileum compared to wild-type mice. Dosh et al. reasoned that this rise in goblet cell number was due to increased expression of the transcription factors, Hath1 and Kruppel-like factor 4 (KLF4) in the inflammatory process (174). The contradictory findings of goblet cell number in IL-10 and IL-1rn deficient mice demonstrate the implications and importance of studying various genetic models reflective of the pathogenesis of intestinal inflammation. Furthermore, genetically altered mice with tamoxifen-induced villin-Cre-dependent intestinal deletion of kindlin 1 and 2 manifest UC-like features due to modified mucus composition and hydrophobicity. These mutant mice develop mucosal colonic inflammation secondary to defective tight junction morphology and extended paracellular space in the mucosal barrier. Here, defective tight junctions prevent the paracellular transport of phosphatidylcholine (PC) causing reduced mucus PC content and a >50% reduction in mucus hydrophobicity. Consequently, the mucosa was predisposed to microbial invasion and subsequent inflammation (175). This finding highlights the role of altered mucus composition as a driving event in UC and not merely a secondary result of inflammation.

In addition to immune markers, recently, significant emphasis has been placed on studying the role of autophagy in mediating intestinal inflammation, and genetically modified animal models have been created to study this phenomenon. Autophagy is an evolutionarily conserved, catabolic cellular mechanism whereby cytoplasmic contents are delivered to, and degraded in, the lysosome (176, 177). A link between autophagy and intestinal inflammation has been proposed, and genome-wide association studies have identified the gene encoding ATG16L1, an autophagy-related protein, as a susceptibility locus for CD (178). Further, mice deficient in the autophagy-related protein, ATG7 in the intestinal epithelial cells (*Atg7*^Δ*IEC*^), have been shown to develop more severe symptoms of colitis when placed on a DSS regimen compared to wild-type mice (179). These mice also had reduced expression of anti-microbial and anti-parasitic peptides, and an increased abundance of gut microbial content. Moreover, evidence suggests that *Atg7*^Δ*IEC*^ mice also have diminished release of intestinal mucins, particularly MUC2, and a less thick mucus layer. These findings indicate that the altered abundance, as well as the increased severity of colitis in *Atg7*^Δ*IEC*^ mice, could at least be partially caused by the reduced levels of intestinal mucins released from goblet cells (179).

Lack of proper glycosylation has been illustrated in UC patients (20, 141, 180) and several genetic models regarding this process provide insight into the implications of altered glycosylation in intestinal inflammation. As mentioned previously, O-linked oligosaccharides, in particular core 1- and core 3- derived mucin type O-glycans, are crucial components in the maintenance and stability of the colonic mucus layer, helping to prevent the penetration of this layer by microbial species via protease degradation and to avert unwarranted activation of the immune response (28, 29, 181). Found throughout the colon, core 1 β1,3-galactosyltransferase (C1GalT1) controls the synthesis of core 1 O-glycans (181). Due to the impaired glycosylation and disrupted mucus integrity found when knocking out this protein's corresponding gene, mice with intestinal epithelium-specific deficiency of core 1 derived O-glycans (*C1galt1*^Δ*IEC*^) develop spontaneous colitis in the distal regions of the colon (181) characterized by myeloid cell infiltration, crypt abscess, epithelial ulceration, goblet cell loss, reduced mucin levels, disrupted mucus layer, and increased epithelial–microbial interaction (153, 180). In contrast to C1GalT1, core 3 β1,3-*N*-acetylglucosaminyltransferase (C3GnT) expression, which regulates core 3 O-glycan formation, is more localized to the proximal colon (181). *C3Gnt*^−/−^ mice show increased susceptibility to colitis and colorectal cancer (182–184). Loss of both intestinal core 1- and 3-derived O-glycans generate mice that develop colitis ranging from the proximal to distal regions of the colon and display earlier onset and more severe intestinal inflammation when compared to *C1galt1*^Δ*IEC*^ mice and *C3Gnt*^−/−^ mice suggesting the loss of these vital components confers compounding deleterious effects (182, 184). Further, the above phenomena with regards to impaired glycosylation of the mucins and its effects on intestinal inflammation seem to be dependent, at least somewhat, on the presence of the resident microbiota; several studies have shown that antibiotic treatment lessens the severity of colitis and boosted mucus layer integrity in these models (180, 181, 183). Thus, evidence from the *C1galt*^Δ*IEC*^, *C3Gnt*^−/−^, and double knockout models suggest proper glycosylation is crucial in maintaining the integrity of the colonic mucus layers, and suggests these O-glycans are a key factor in protecting the underlying epithelium from abnormal microbial interaction and preventing unsolicited intestinal inflammation.

From the above evidence, it is clear that within different physiological environments, different mucins, and different alterations in those mucins can greatly affect the host susceptibility to intestinal inflammation. These findings highlight the essential role of mucin in the pathogenesis of intestinal inflammation and exemplify the importance of maintaining mucin levels within a healthy homeostatic range. Because of this complexity, further research must be carried out to clearly elucidate the various roles of mucins in IBD.

### Enteric Infection

*Trichuris trichiura* is a soil-transmitted helminth which affects millions of people, particularly children, around the world (185). To gain a more comprehensive understanding of the immune response against *T. trichiura*, enteric parasitic animal models have been developed. One such model which has been extensively used in both our laboratory and others is the murine infection, *T. muris*. Resistant mice or those that are able to expel worms successfully display a characteristic Th2 immune response with the upregulation of IL-4 and IL-13. In contrast, susceptible hosts such as AKR mice develop a Th1 immune response associated with chronic infection and increased worm burden (186). Changes in mucin production, both qualitative and quantitative, can affect host protection against parasitic infections, and thus, the protective role of mucins in *T. muris* infection is increasingly being explored.

There are several methods through which mucins contribute to parasitic clearance. Firstly, it has been shown that some mucins, such as MUC5AC, have direct damaging effects on worms and reduce their viability (31). Thus, it comes as no surprise that mice deficient in MUC2 or MUC5AC show delayed worm expulsion in response to *T. muris* infection (31, 187). Secondly, mucins can create a thick and impermeable physical barrier to protect the underlying epithelial cells from *T. muris* invasion. In fact, *T. muris* infection leads to an increase in the thickness of glycocalyx, particularly due to the upregulation of MUC4, MUC13, and MUC17 proteins (103). Interestingly, the serine proteases secreted by *T. muris* can degrade MUC2, but not MUC5AC, giving this mucin considerable influence on the successful clearance of these invaders (31, 188).

*Entamoeba histolytica*, a human protozoan parasite, causes amebic colitis, and liver abscess, a condition collectively called amebiasis (189). Mostly present in the developing world, symptoms manifest in 10% of infected individuals possibly due to variation in host immune response (189). Interestingly, MUC2 plays a vital role in the development and progression of amebiasis (189). After ingestion of contaminated food or water*, E. histolytica* colonizes the colonic outer mucus layer by binding to glycans on the MUC2 molecule via its Gal/GalNAc-lectin (190). The protozoa then cleave MUC2 by glycosidases and proteases and subsequently, comes in to contact with intestinal epithelial cells (191, 192). Thus, the strength of the mucin layer is a key player in the physical defense against *E. histolytica*-induced inflammation and epithelial invasion. In addition to MUC2 degradation, altered MUC2 production is found in intestinal amebiasis. *E. histolytica* has been found to bind to αvβ3 integrins on goblet cells and stimulate the hypersecretion of mucus by exocytosis (193). Contrasting data suggests, however, that *E. histolytica* impairs the regulation of Math1 transcription factor required for goblet cell differentiation and can actually lead to decreased mucus production (194). In addition, during *E. histolytica* infection, MUC2, possibly by acting either as a cAMP ligand or by activating TLRs, can promote elevated levels of antimicrobial peptides such as cathelicidins (195).

*Clostridium difficile* is an anaerobic, spore-forming bacterium that causes worldwide epidemics with significant mortality rates (196). Initial infection by this enteric pathogen is characterized by an acute inflammatory response with a substantial influx of neutrophils, diarrhea, and weight loss (197). Fortunately, murine colitis models utilizing *C*. *difficile* have provided a better understanding of this infection. Previously, it has been shown that patients with *C*. *difficile* infection secreted acidic mucus primarily composed of MUC1 and have decreased MUC2 expression indicating defective mucosal barrier function (198). Moreover, patients with *C. difficile* infection exhibited altered mucus composition with higher GLcNAc and galactose levels, but lower GalNAc levels (198). Intriguingly, fecal microbiota transplantation (FMT) has received a growing interest as an effective therapeutic strategy to treat this infection. Recently, it has been shown that upon IL-33 treatment, *C. difficile-*infected mice exhibited increased goblet cell number and mucin production via activation of IL-13-secreting ILC2s and FMT rescued IL-33 expression in the colon after antibiotic-mediated depletion (199). Other enteric bacterial infections have also illustrated the implications of alteration within the mucus layer. For instance, animal studies have shown that deficiency in the transmembrane mucin, MUC1, results in increased susceptibility and more severe intestinal damage in response to infection with *Campylobacter jejuni* (200). Similarly, mice deficient in MUC2, the main ingredient of colonic mucus, had increased intestinal permeability and were more susceptible to *S. enterica* serovar Typhimurium infection (201, 202).

In addition to alteration in quantity, post-translational modifications can also affect the ability of mucin to maintain intestinal barrier integrity. One such modification, the addition of fatty acids called palmitoylation, is particularly important for maintaining this barrier function. Inactivation of the enzyme, fatty acid synthase (FAS) in intestinal epithelial cells prevents palmitoylation of MUC2, and ultimately leads to an increase in intestinal permeability (203, 204). Improper sulfonation, or the addition of sulfate anions to mucins, has similar effects of intestinal permeability in *C. jejuni* infection (205). In addition, barrier function and pathogen adhesion are largely regulated by the diverse glycosylation pattern of mucins. Analysis of gastric mucin glycosylation profiles reveal important roles of terminal α1,2-fucose residues on Lewis-b and H type 1 structures expressed on MUC5AC (206). By utilizing α1,2-fucosyltransferase-deficient (*FUT2*^−/−^*)* mice, it has been established that the binding capacity of the infectious agent, *Helicobacter pylori*, to Lewis-b and H-type antigens is impaired due to the loss of gastric MUC5AC fucosylation (207). Similarly, norovirus infection is dependent on the fucosylation status of soluble mucins within the GI tract (206, 208). It is speculated that reduced mucin fucosylation in the GI tract results in decreased mucus thickness and increased intestinal permeability to pathogens (206). Further, inhibition of the enzyme, core 3 β1,3-N-acetylglucosaminyltransferase (C3GnT) which plays a crucial role in the addition of O-linked oligosaccharides, can alter the structural and functional features of mucins and has been shown to decrease MUC2 levels and increase intestinal permeability (184, 209). Moreover, goblet cells in mice resistant to chronic *T. muris* infection contain high levels of sulphated mucins in contrast with the goblet cells of susceptible mice which are dominated by sialylated mucins. Consequently, sulphation of mucins promoted by Th2 immune mediators, such as IL-13, augments the protection offered by mucins by aiding in their ability to resist degradation (210).

Table 1 summarizes the various and dynamic changes in mucins and the mucus layer in the context of the aforementioned pathologies and animal models.

**Table 1:** The intestinal mucus layer is a dynamic part of the innate immune system which undergoes quantitative and qualitative changes in response to inflammation. Evidence collected in clinical studies, as well as information gained from animal experiments, have shed light on some of these changes.

	**Condition**	**Effects observed**	**References**
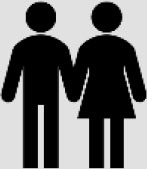	Crohn's disease	↑Mucus thickness or no change ↓MUC2, MUC3, MUC4, MUC5B, MUC7 MUC5AC and MUC6 present Goblet cell depletion	(145, 146) (155, 156) (155) (144)
	Ulcerative colitis	↓Mucus thickness ↓Glycosylation and sulphation ↑Sialylation ↓MUC2, MUC9, MUC20 ↑MUC1, MUC16 MUC5AC present Goblet cell depletion	(145, 146) (20) (147, 150, 151) (149, 151) (148) (144)
	Colorectal cancer	↑MUC1 ↓MUC2 *De novo* MUC5AC synthesis	(158) (12, 161) (162, 163)
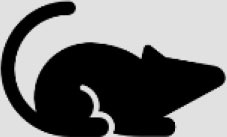	Chemical colitis	↑MUC2 (or no change) ↑MUC2 followed by rapid reduction ↑Th1 cytokines (IL-12, TNF-α etc.)	(166) (167) (165)
	Enteric parasitic infection	↑Th2 cytokines (IL-4 and IL-13 etc.) ↑Mucin production/goblet cell hyperplasia ↑Thickness of glycocalyx/MUC4, MUC13, MUC17	(97, 98, 186) (98–103) (103)
	Genetic models:		
	1) *Muc4^−/−^* 2) *Muc2^−/−^* 3) *Atg7^Δ*IEC*^* 4) *IL-1rn^−/−^* *5) C1galt1^Δ*IEC*^*	↓DSS inflammation severity ↑MUC2 and MUC3 ↑DSS inflammation severity/spontaneous colitis ↑DSS inflammation severity ↓MUC2/mucus layer thickness ↑Goblet cell number Spontaneous colitis Microbially breached mucus layer	(170) (169) (179) (174) (153, 180, 181)

## Discussion

In the current review, we have highlighted the structural and functional features as well as the immunological regulation of mucins and have examined, within the context of intestinal inflammation, the changes associated in goblet cell biology and mucin production. The collection of evidence herein gives credence not only to the significant role mucins play in barrier function but also to the bidirectional relationship between mucus production and the immune system.

Several inferences can be made from the evidence presented. Firstly, the intestinal mucus layer is a highly dynamic system which responds to various pathological alterations within the GI tract, including changes in enteric infection, colorectal cancer, and IBD. While examining the evidence, it was noted that much of the research investigating the role of mucins in intestinal inflammation focuses on animal models and enteric infection. Hence, further clinical research would promote a better understanding of the role of mucin in these pathogeneses and also potentially open up new avenues of treatment. Secondly, dysfunction, whether qualitative or quantitative, of the mucus layer causes a sharp reduction in its ability to maintain barrier function. The presented evidence has highlighted the critical role of mucins in offering protection in the context of intestinal inflammation. The conflicting results with respect to several gel-forming and transmembrane mucin knockout models such as *Muc2*^−/−^ and *Muc4*^−/−^ illustrate that not all mucins confer similar effects within the context of colitis. Inability to maintain a delicate balance of the proper ratios and varieties of mucins, thus, can significantly affect the host susceptibility to intestinal inflammation. Due to this complexity, more research must be done to further clarify the various roles of mucin in IBD pathogenesis. Similarly, the above analysis has illustrated that post-translational modifications such as sialylation, sulphation, and O-glycosylation are altered in response to several pathological conditions and can greatly alter the functional properties of the mucus layer. Investigating if reversing or supplementing the effects of these altered post-translational modifications also attenuates disease severity will prove interesting. Thirdly, via TLRs, NLRs, ILCs, and T lymphocytes through several Th2 cytokines, the immune system can significantly influence the production of mucins and the quality and efficacy these mucins display in maintaining the gut barrier. Though not exclusively touched on in this review, the impact pathogenic organisms have on the resident gut microbial community and how these changes influence goblet cell function and mucin production will provide an interesting area for further exploration.

Above all, evidence from clinical and animal models profoundly suggests that alterations in the mucus layer, aberrant post-translational modifications, and differential expression of key mucins are critical factors in the pathogenesis and severity of several conditions including enteric infection, colorectal cancer, and IBD, further emphasizing the importance of maintaining mucin levels within a healthy homeostatic range. Future studies on the impact of mucins within these conditions can only further our understanding of the immunological regulation and clinical implications of mucins within the GI tract.

## Author Contributions

JG, YK, PF, and SH reviewed the literature and wrote the manuscript. JG designed and created the figures. JG, YK, and WK edited and revised the manuscript. PF and YK conceived the idea for the article. WK supervised the project. All authors provided critical feedback and shaped the final manuscript.

## Conflict of Interest

The authors declare that the research was conducted in the absence of any commercial or financial relationships that could be construed as a potential conflict of interest.

## Abbreviations

AMP: antimicrobial peptides
IgA: immunoglobulin-A
IL: interleukin
TNF-α: tumor necrosis factor alpha
GalNAc: N-acetyl galactosamine
GlcNAc: N-acetyl glucosamine
Gal: galactose
Fuc: fucose
TLRs: Toll-like receptors
SPDEF: SAM pointed domain-containing ETS transcription factor
NLRs: nucleotide-binding oligomerization domain (NOD)-like receptors
LPS: lipopolysaccharide
LTA: lipoteichoic acid
PAMPs: pathogen associated molecular patterns
LBP: lipopolysaccharide (LPS) binding protein
NF-κB: nuclear factor kappa-light-chain-enhancer of activated B cells
ASGM1: asialoGM1
ILC: innate lymphoid cell
CLP: common lymphoid progenitor
RORα: retinoic acid receptor-related orphan receptor alpha
GATA3: GATA binding protein 3
Ach: acetylcholine
MR: muscarinic acetylcholine receptors
NR: nicotinic acetylcholine receptor, M3R, type 3 muscarinic receptor
VIP: vasoactive intestinal peptide
VPAC: vasoactive intestinal peptide (VIP) receptor
Chrm3: cholinergic receptor muscarinic 3
NmU: neuromedin U
GWAS: genome-wide association study
FCGBP: IgGFc-binding protein
CLCA1: calcium-activated chloride channel regulator 1
ZG16: zymogen granule protein 16
ESPs: excretory/secretory products
Nmur1: neuromedin U (NmU) receptor 1
LRRK2: leucine-rich repeat kinase 2
KLF4: Kruppel-like factor 4
PC: phosphatidylcholine
C1GalT1: core 1 β1,3-galactosyltransferase
C3GnT: core 3 β1,3-N-acetylglucosaminyltransferase
FMT: fecal microbiota transplantation
FAS: fatty acid synthase.

## References

[1] Vighi G Marcucci F Sensi L Di Cara G Frati F. Allergy and the gastrointestinal system. Clin Exp Immunol. (2008) 153:3–6. 10.1111/j.1365-2249.2008.03713.x 18721321 PMC2515351

[2] Lamont JT. Mucus: the front line of intestinal mucosal defense. Ann N Y Acad Sci. (1992) 664:190–201. 10.1111/j.1749-6632.1992.tb39760.x 1456650

[3] Kim J Khan W. Goblet cells and mucins: role in innate defense in enteric infections. Pathogens. (2013) 2:55–70. 10.3390/pathogens2010055 25436881 PMC4235714

[4] Van Putten JPM Strijbis K. Transmembrane mucins: signaling receptors at the intersection of inflammation and cancer. J Innate Immun. (2017) 9:281–99. 10.1159/000453594 28052300 PMC5516414

[5] Carraway KL Ramsauer VP Haq B Carothers Carraway CA. Cell signaling through membrane mucins. BioEssays. (2003) 25:66–71. 10.1002/bies.10201 12508284

[6] Singh PK Hollingsworth MA. Cell surface-associated mucins in signal transduction. Trends Cell Biol. (2006) 16:467–76. 10.1016/j.tcb.2006.07.006 16904320

[7] Strugnell RA Wijburg OLC. The role of secretory antibodies in infection immunity. Nat Rev Microbiol. (2010) 8:656–67. 10.1038/nrmicro2384 20694027

[8] Atuma C Strugala V Allen A Holm L. The adherent gastrointestinal mucus gel layer: thickness and physical state *in vivo*. Am J Physiol Gastrointest Liver Physiol. (2001) 280:G922–9. 10.1152/ajpgi.2001.280.5.g922 11292601

[9] Hansson GC. Role of mucus layers in gut infection and inflammation. Curr Opin Microbiol. (2012) 15:57–62. 10.1016/j.mib.2011.11.002 22177113 PMC3716454

[10] Gordon JI Schmidt GH Roth KA. Studies of intestinal stem cells using normal, chimeric, and transgenic mice. FASEB J. (1992) 6:3039–50. 10.1096/fasebj.6.12.1521737 1521737

[11] Johansson MEV Hansson GC. Immunological aspects of intestinal mucus and mucins. Nat Rev Immunol. (2016) 16:639–49. 10.1038/nri.2016.88 27498766 PMC6435297

[12] Weiss AA Babyatsky MW Ogata S Chen A Itzkowitz SH. Expression of MUC2 and MUC3 mRNA in human normal, malignant, and inflammatory intestinal tissues. J Histochem Cytochem. (1996) 44:1161–6. 10.1177/44.10.8813081 8813081

[13] Akiba Y Guth PH Engel E Nastaskin I Kaunitz JD. Dynamic regulation of mucus gel thickness in rat duodenum. Am J Physiol Gastrointest Liver Physiol. (2000) 279:G437–47. 10.1016/S0016-5085(00)85585-7 10915654

[14] Specian RD Oliver MG Functional biology of intestinal goblet cells. Am J Physiol. (1991) 260:C183–93. 10.1152/ajpcell.1991.260.2.C183 1996606

[15] Zalewsky CA Moody FG. Mechanisms of mucus release in exposed canine gastric mucosa. Gastroenterology. (1979) 77:719–29. 10.1016/0016-5085(79)90228-2 467928

[16] Helander HF. The cells of the gastric mucosa. Int Rev Cytol. (1981) 70:217–89. 10.1016/S0074-7696(08)61133-X 7014505

[17] Ito S Winchester RJ. The fine structure of the gastric mucosa in the bat. J Cell Biol. (1963) 16:541–77. 10.1083/jcb.16.3.541 13957001 PMC2106229

[18] Helander HF Ultrastructure of fundus glands of the mouse gastric mucosa. J Ultrastruct Res. (1962) 7:1–123. 10.1016/s0022-5320(62)80047-1 13906170

[19] Mills JC Shivdasani RA. Gastric epithelial stem cells. Gastroenterology. (2011) 140:412–4. 10.1053/j.gastro.2010.12.001 21144849 PMC3708552

[20] Boltin D Perets TT Vilkin A Niv Y. Mucin function in inflammatory bowel disease: an update. J Clin Gastroenterol. (2013) 47:106–11. 10.1097/MCG.0b013e3182688e73 23164684

[21] Khan WI. Physiological changes in the gastrointestinal tract and host protective immunity: learning from the mouse-Trichinella spiralis model. Parasitology. (2008) 135:671–82. 10.1017/S0031182008004381 18501042

[22] Kaser A Zeissig S Blumberg RS Inflammatory bowel disease. Annu Rev Immunol. (2010) 28:573–621. 10.1146/annurev-immunol-030409-101225 20192811 PMC4620040

[23] Kaplan GG. The global burden of IBD: From 2015 to 2025. Nat Rev Gastroenterol Hepatol. (2015) 12:720–7. 10.1038/nrgastro.2015.150 26323879

[24] Petri WA Miller M Binder HJ Levine MM Dillingham R Guerrant RL. Enteric infections, diarrhea, and their impact on function and development. J Clin Invest. (2008) 118:1277–90. 10.1172/JCI34005 18382740 PMC2276781

[25] Dharmani P Srivastava V Kissoon-Singh V Chadee K. Role of intestinal mucins in innate host defense mechanisms against pathogens. J Innate Immun. (2009) 1:123–35. 10.1159/000163037 20375571 PMC7312850

[26] Linden SK Sutton P Karlsson NG Korolik V McGuckin MA. Mucins in the mucosal barrier to infection. Mucosal Immunol. (2008) 1:183–97. 10.1038/mi.2008.5 19079178 PMC7100821

[27] Rao C V Janakiram NB Mohammed A. Molecular pathways molecular pathways: mucins and drug delivery in cancer. (2016). 10.1158/1078-0432.CCR-16-0862 28039261 PMC6038927

[28] Devine PL McKenzie IFC. Mucins: structure, function, and associations with malignancy. BioEssays. (1992) 14:619–25. 10.1002/bies.950140909 1365918

[29] Tran DT Ten Hagen KG. Mucin-type o-glycosylation during development. J Biol Chem. (2013) 288:6921–9. 10.1074/jbc.R112.418558 23329828 PMC3591602

[30] Corfield AP. Mucins: a biologically relevant glycan barrier in mucosal protection. Biochim Biophys Acta Gen Sub. (2015) 1850:236–52. 10.1016/j.bbagen.2014.05.003 24821013

[31] Hasnain SZ Evans CM Roy M Gallagher AL Kindrachuk KN Barron L. Muc5ac: a critical component mediating the rejection of enteric nematodes. J Exp Med. (2011) 208:893–900. 10.1084/jem.20102057 21502330 PMC3092342

[32] Reid CJ Harris A. Expression of the MUC 6 mucin gene in development of the human kidney and male genital ducts. J Histochem Cytochem. (1999) 47:817–21. 10.1177/002215549904700611 10330458

[33] Walsh MD Clendenning M Williamson E Pearson SA Walters RJ Nagler B. Expression of MUC2, MUC5AC, MUC5B, and MUC6 mucins in colorectal cancers and their association with the CpG island methylator phenotype. Mod Pathol. (2013) 26:1642–56. 10.1038/modpathol.2013.101 23807779

[34] Li S Bobek LA. Functional analysis of human MUC7 mucin gene 5′-flanking region in lung epithelial cells. Am J Respir Cell Mol Biol. (2006) 35:593–601. 10.1165/rcmb.2006-0110OC 16778149 PMC2643277

[35] Frenkel ES Ribbeck K. Salivary mucins in host defense and disease prevention. J Oral Microbiol. (2015) 7:29759. 10.3402/jom.v7.29759 26701274 PMC4689954

[36] Schneider H Pelaseyed T Svensson F Johansson MEV. Study of mucin turnover in the small intestine by *in vivo* labeling. Sci Rep. (2018) 8:5760. 10.1038/s41598-018-24148-x 29636525 PMC5893601

[37] Tailford LE Crost EH Kavanaugh D Juge N. Mucin glycan foraging in the human gut microbiome. Front Genet. (2015) 5:81. 10.3389/fgene.2015.00081 25852737 PMC4365749

[38] Kim YS Ho SB. Intestinal goblet cells and mucins in health and disease: recent insights and progress. Curr Gastroenterol Rep. (2010) 12:319–330. 10.1007/s11894-010-0131-2 20703838 PMC2933006

[39] Hattrup CL Gendler SJ. Structure and function of the cell surface (tethered) mucins. Annu Rev Physiol. (2008) 70:431–57. 10.1146/annurev.physiol.70.113006.100659 17850209

[40] Kato K Lillehoj EP Lu W Kim KC. MUC1: the first respiratory mucin with an anti-inflammatory function. J Clin Med. (2017) 6:110. 10.3390/jcm6120110 29186029 PMC5742799

[41] Zecchini V Domaschenz R Winton D Jones P. Notch signaling regulates the differentiation of post-mitotic intestinal epithelial cells. Genes Dev. (2005) 19:1686–91. 10.1101/gad.341705 16024658 PMC1176006

[42] Kopan R Notch signaling. Cold Spring Harb Perspect Biol. (2012) 4:a011213 10.1101/cshperspect.a011213 23028119 PMC3475170

[43] Zheng H Pritchard DM Yang X Bennett E Liu G Liu C. KLF4 gene expression is inhibited by the notch signaling pathway that controls goblet cell differentiation in mouse gastrointestinal tract. Am J Physiol Liver Physiol. (2009) 296:G490–8. 10.1152/ajpgi.90393.2008 19109406 PMC2660173

[44] Van Es JH Van Gijn ME Riccio O Van Den Born M Vooijs M Begthel H. Notch/γ-secretase inhibition turns proliferative cells in intestinal crypts and adenomas into goblet cells. Nature. (2005) 435:959–63. 10.1038/nature03659 15959515

[45] Radwan KA Oliver MG Specian RD. Cytoarchitectural reorganization of rabbit colonic goblet cells during baseline secretion. Am J Anat. (1990) 189:365–76. 10.1002/aja.1001890408 2285043

[46] Noah TK Kazanjian A Whitsett J Shroyer NF. SAM pointed domain ETS factor (SPDEF) regulates terminal differentiation and maturation of intestinal goblet cells. Exp Cell Res. (2010) 316:452–65. 10.1016/j.yexcr.2009.09.020 19786015 PMC3004755

[47] Specian RD Neutra MR. Cytoskeleton of intestinal goblet cells in rabbit and monkey. The theca. Gastroenterology. (1984) 87:1313–25. 10.1016/0016-5085(84)90198-7 6541604

[48] Welsey A Mantle M Man D Qureshi R. Neutral and acidic species of human intestinal mucin. J Biol Chem. (1985) 260:7955–9. 4008485

[49] Phillips TE Phillips TH Neutra MR. Regulation of intestinal goblet cell secretion. III. Isolated intestinal epithelium. Am J Physiol. (1984) 247(6 Pt 1):G674–81. 10.1152/ajpgi.1984.247.6.g674 6391203

[50] Gum JR Hicks JW Gillespie A-M Carlson EJ Kömüves L Karnik S. Goblet cell-specific expression mediated by the *MUC2* mucin gene promoter in the intestine of transgenic mice. Am J Physiol Liver Physiol. (1999) 276:G666–76. 10.1152/ajpgi.1999.276.3.G666 10070043

[51] Forstner G. Signal transduction packaging and secretion of mucins. Annu Rev Physiol. (1995) 57:585–605. 10.1146/annurev.ph.57.030195.003101 7778879

[52] Villalobo A Gabius H-J. Signaling pathways for transduction of the initial message of the glycocode into cellular responses. Cells Tissues Organs. (1998) 161:110–29. 10.1159/000046453 9780354

[53] Specian RD Neutra MR. Mechanism of rapid mucus secretion in goblet cells stimulated by acetylcholine. J Cell Biol. (1980) 85:626–40. 10.1083/jcb.85.3.626 7391135 PMC2111470

[54] Birchenough GMH Nystrom EEL Johansson MEV Hansson GC. A sentinel goblet cell guards the colonic crypt by triggering Nlrp6-dependent Muc2 secretion. Science. (2016) 352:1535–42. 10.1126/science.aaf7419 27339979 PMC5148821

[55] Chaplin DD. Overview of the immune response. J Allergy Clin Immunol. (2010) 125(2 Suppl. 2):S3–23. 10.1016/j.jaci.2009.12.980 20176265 PMC2923430

[56] Mogensen TH. Pathogen recognition and inflammatory signaling in innate immune defenses. Clin Microbiol Rev. (2009) 22:240–73. 10.1128/CMR.00046-08 19366914 PMC2668232

[57] Paul WE. Bridging innate and adaptive immunity. Cell. (2011) 147:1212–5. 10.1016/j.cell.2011.11.036 22153065

[58] Kelly D Conway S Aminov R. Commensal gut bacteria: mechanisms of immune modulation. Trends Immunol. (2005) 26:326–33. 10.1016/j.it.2005.04.008 15922949

[59] McClure R Massari P. TLR-dependent human mucosal epithelial cell responses to microbial pathogens. Front Immunol. (2014) 5:386. 10.3389/fimmu.2014.00386 25161655 PMC4129373

[60] Price AE Shamardani K Lugo KA Deguine J Roberts AW Lee BL. A map of Toll-like receptor expression in the intestinal epithelium reveals distinct spatial, cell type-specific, and temporal patterns. Immunity. (2018) 49:560–75.e6. 10.1016/j.immuni.2018.07.016 30170812 PMC6152941

[61] Burgueño JF Abreu MT. Epithelial Toll-like receptors and their role in gut homeostasis and disease. Nat Rev Gastroenterol Hepatol. (2020) 17:263–78. 10.1038/s41575-019-0261-4 32103203

[62] Hayashi F Smith KD Ozinsky A Hawn TR Yi EC Goodlett DR. The innate immune response to bacterial flagellin is mediated by Toll- like receptor 5. Nature. (2001) 410:1099–3. 10.1038/35074106 11323673

[63] Wlodarska M Thaiss CA Nowarski R Henao-Mejia J Zhang JP Brown EM. NLRP6 inflammasome orchestrates the colonic host-microbial interface by regulating goblet cell mucus secretion. Cell. (2014) 156:1045–59. 10.1016/j.cell.2014.01.026 24581500 PMC4017640

[64] Khan MA Ma C Knodler LA Valdez Y Rosenberger CM Deng W. Toll-like receptor 4 contributes to colitis development but not to host defense during *Citrobacter rodentium* infection in mice. Infect Immun. (2006) 74:2522–36. 10.1128/IAI.74.5.2522-2536.2006 16622187 PMC1459750

[65] Sodhi CP Neal MD Siggers R Sho S Ma C Branca MF. Intestinal epithelial toll-like receptor 4 regulates goblet cell development and is required for necrotizing enterocolitis in mice. Gastroenterology. (2012) 143:708–18.e5. 10.1053/j.gastro.2012.05.053 22796522 PMC3584415

[66] McNamara N Basbaum C. Signaling networks controlling mucin production in response to Gram-positive and Gram-negative bacteria. Glycoconj J. (2001) 18:715–22. 10.1023/A:1020875423678 12386457

[67] Kamdar K Johnson AMF Chac D Myers K Kulur V Truevillian K. Innate recognition of the microbiota by TLR1 promotes epithelial homeostasis and prevents chronic inflammation. J Immunol. (2018) 201:230–42. 10.4049/jimmunol.1701216 29794015 PMC6903428

[68] Carvalho FA Koren O Goodrich JK Johansson MEV Nalbantoglu I Aitken JD. Transient inability to manage proteobacteria promotes chronic gut inflammation in TLR5-deficient mice. Cell Host Microbe. (2012) 12:139–52. 10.1016/j.chom.2012.07.004 22863420 PMC4310462

[69] Lee K-D Guk S-M Chai J-Y. Toll-like receptor 2 and Muc2 expression on human intestinal epithelial cells by Gymnophalloides seoi adult antigen. J Parasitol. (2010) 96:58–66. 10.1645/GE-2195.1 19737027

[70] Oviedo-Boyso J Bravo-Patiño A Baizabal-Aguirre VM. Collaborative action of toll-like and nod-like receptors as modulators of the inflammatory response to pathogenic bacteria. Mediators Inflamm. (2014) 2014:432785. 10.1155/2014/432785 25525300 PMC4267164

[71] Moreira LO Zamboni DS. NOD1 and NOD2 signaling in infection and inflammation. Front Immunol. (2012) 3:328. 10.3389/fimmu.2012.00328 23162548 PMC3492658

[72] Wang H Kim JJ Denou E Gallagher A Thornton DJ Shajib MS. New role of nod proteins in regulation of intestinal goblet cell response in the context of innate host defense in an enteric parasite infection. Infect Immun. (2016) 84:275–85. 10.1128/IAI.01187-15 26527214 PMC4694016

[73] McNamara N Khong A McKemy D Caterina M Boyer J Julius D. ATP transduces signals from ASGM1, a glycolipid that functions as a bacterial receptor. Proc Natl Acad Sci USA. (2001) 98:9086–91. 10.1073/pnas.161290898 11481474 PMC55377

[74] Gurram RK Zhu J. Orchestration between ILC2s and Th2 cells in shaping type 2 immune responses. Cell Mol Immunol. (2019) 16:225–35. 10.1038/s41423-019-0210-8 30792500 PMC6460501

[75] Van Dyken SJ Locksley RM. Interleukin-4- and interleukin-13-mediated alternatively activated macrophages: roles in homeostasis and disease. Annu Rev Immunol. (2013) 31:317–43. 10.1146/annurev-immunol-032712-095906 23298208 PMC3606684

[76] Tu L Chen J Xu D Xie Z Yu B Tao Y. IL-33-induced alternatively activated macrophage attenuates the development of TNBS-induced colitis. Oncotarget. (2017) 8:27704–14. 10.18632/oncotarget.15984 28423665 PMC5438602

[77] Withers DR. Innate lymphoid cell regulation of adaptive immunity. Immunology. (2016) 149:123–30. 10.1111/imm.12639 27341319 PMC5011676

[78] Artis D Spits H. The biology of innate lymphoid cells. Nature. (2015) 517:293–301. 10.1038/nature14189 25592534

[79] Goto Y Obata T Kunisawa J Sato S Ivanov II Lamichhane A. Innate lymphoid cells regulate intestinal epithelial cell glycosylation. Science. (2014) 345: 10.1126/science.1254009 25214634 PMC4774895

[80] Klose CSN Kiss EA Schwierzeck V Ebert K Hoyler T D'Hargues Y. A T-bet gradient controls the fate and function of CCR6-RORγt + innate lymphoid cells. Nature. (2013) 494:261–5. 10.1038/nature11813 23334414

[81] Serafini N Vosshenrich CAJ Di Santo JP. Transcriptional regulation of innate lymphoid cell fate. Nat Rev Immunol. (2015) 15:415–28. 10.1038/nri3855 26065585

[82] Tait Wojno ED Artis D. Emerging concepts and future challenges in innate lymphoid cell biology. J Exp Med. (2016) 213:2229–48. 10.1084/jem.20160525 27811053 PMC5068238

[83] Wilhelm C Hirota K Stieglitz B Van Snick J Tolaini M Lahl K. An IL-9 fate reporter demonstrates the induction of an innate IL-9 response in lung inflammation. Nat Immunol. (2011) 12:1071–7. 10.1038/ni.2133 21983833 PMC3198843

[84] Moro K Yamada T Tanabe M Takeuchi T Ikawa T Kawamoto H. Innate production of TH 2 cytokines by adipose tissue-associated c-Kit+ Sca-1+ lymphoid cells. Nature. (2010) 463:540–4. 10.1038/nature08636 20023630

[85] Neill DR Wong SH Bellosi A Flynn RJ Daly M Langford TKA. Nuocytes represent a new innate effector leukocyte that mediates type-2 immunity. Nature. (2010) 464:1367–70. 10.1038/nature08900 20200518 PMC2862165

[86] Price AE Liang HE Sullivan BM Reinhardt RL Eisley CJ Erle DJ. Systemically dispersed innate IL-13-expressing cells in type 2 immunity. Proc Natl Acad Sci USA. (2010) 107:11489–94. 10.1073/pnas.1003988107 20534524 PMC2895098

[87] Waddell A Vallance JE Hummel A Alenghat T Rosen MJ. IL-33 induces murine intestinal goblet cell differentiation indirectly via innate lymphoid cell IL-13 secretion. J Immunol. (2019) 202:598–607. 10.4049/jimmunol.1800292 30530480 PMC6324976

[88] Hung LY Lewkowich IP Dawson LA Downey J Yang Y Smith DE. IL-33 drives biphasic IL-13 production for noncanonical Type 2 immunity against hookworms. Proc Natl Acad Sci USA. (2013) 110:282–7. 10.1073/pnas.1206587110 23248269 PMC3538196

[89] Klose CSN Artis D. Innate lymphoid cells as regulators of immunity, inflammation and tissue homeostasis. Nat Immunol. (2016) 17:765–74. 10.1038/ni.3489 27328006

[90] Zeng B Shi S Ashworth G Dong C Liu J Xing F. ILC3 function as a double-edged sword in inflammatory bowel diseases. Cell Death Dis. (2019) 10:1–12. 10.1038/s41419-019-1540-2 30962426 PMC6453898

[91] Sonnenberg GF Fouser LA Artis D. Border patrol: regulation of immunity, inflammation and tissue homeostasis at barrier surfaces by IL-22. Nat Immunol. (2011) 12:383–90. 10.1038/ni.2025 21502992

[92] Philpott DJ Sorbara MT Robertson SJ Croitoru K Girardin SE. NOD proteins: regulators of inflammation in health and disease. Nat Rev Immunol. (2014) 14:9–23. 10.1038/nri3565 24336102

[93] Bonilla FA Oettgen HC. Adaptive immunity. J Allergy Clin Immunol. (2010) 125(2 Suppl. 2):S33–40. 10.1016/j.jaci.2009.09.017 20061006

[94] Kayama H Takeda K. Regulation of intestinal homeostasis by innate and adaptive immunity. Int Immunol. (2012) 24:673–80. 10.1093/intimm/dxs094 22962437

[95] Enss ML Cornberg M Wagner S Gebert A Henrichs M Eisenblätter R. Proinflammatory cytokines trigger MUC gene expression and mucin release in the intestinal cancer cell line LS180. Inflamm Res. (2000) 49:162–9. 10.1007/s000110050576 10858016

[96] Khan WI Abe T Ishikawa N Nawa Y Yoshimura K. Reduced amount of intestinal mucus by treatment with anti-CD4 antibody interferes with the spontaneous cure of *Nippostrongylus brasiliensis*-infection in mice. Parasite Immunol. (1995) 17:485–91. 10.1111/j.1365-3024.1995.tb00919.x 8552418

[97] Grencis RK. Th2-mediated host protective immunity to intestinal nematode infections. Philos Trans R Soc B Biol Sci. (1997) 352:1377–84. 10.1098/rstb.1997.0123 9355130 PMC1692029

[98] Else KJ Finkelman FD. Intestinal nematode parasites, cytokines and effector mechanisms. Int J Parasitol. (1998) 28:1145–58. 10.1016/S0020-7519(98)00087-3 9762559

[99] Miller HRP Nawa Y. Nippostrongylus brasiliensis: intestinal goblet-cell response in adoptively immunized rats. Exp Parasitol. (1979) 47:81–90. 10.1016/0014-4894(79)90010-9 421768

[100] Carroll SM Mayrhofer G Dawkins HJS Grove DI. Kinetics of intestinal lamina propria mast cells, globule leucocytes, intraepithelial lymphocytes, goblet cells and eosinophils in murine strongyloidiasis. Int Arch Allergy Immunol. (1984) 74:311–7. 10.1159/000233566 6735488

[101] Ishikawa N Wakelin D Mahida YR. Role of T helper 2 cells in intestinal goblet cell hyperplasia in mice infected with *Trichinella spiralis*. Gastroenterology. (1997) 113:542–9. 10.1053/gast.1997.v113.pm9247474 9247474

[102] Oeser K Schwartz C Voehringer D. Conditional IL-4/IL-13-deficient mice reveal a critical role of innate immune cells for protective immunity against gastrointestinal helminths. Mucosal Immunol. (2015) 8:672–82. 10.1038/mi.2014.101 25336167

[103] Hasnain SZ Thorton DJ Grencis RK. Changes in the mucosal barrier during acute and chronic *Trichuris muris* infection. Parasite Immunol. (2011) 33:45–55. 10.1111/j.1365-3024.2010.01258.x 21155842 PMC3020324

[104] Khan WI Blennerhasset P Ma C Matthaei KI Collins SM. Stat6 dependent goblet cell hyperplasia during intestinal nematode infection. Parasite Immunol. (2001) 23:39–42. 10.1046/j.1365-3024.2001.00353.x 11136476

[105] Mckay DM Khan Waliul I. STAT-6 is an absolute requirement for murine rejection of *Hymenolepis diminuta*. Source J Parasitol. 89:188–9. 10.1645/0022-3395(2003)089[0188:SIAARF]2.0.CO;2 12659328

[106] Marillier RG Michels C Smith EM Fick LC Leeto M Dewals B. IL-4/IL-13 independent goblet cell hyperplasia in experimental helminth infections. BMC Immunol. (2008) 9:11. 10.1186/1471-2172-9-11 18373844 PMC2329604

[107] Pelly VS Kannan Y Coomes SM Entwistle LJ Rückerl D Seddon B. IL-4-producing ILC2s are required for the differentiation of TH2 cells following Heligmosomoides polygyrus infection. Mucosal Immunol. (2016) 9:1407–17. 10.1038/mi.2016.4 26883724 PMC5257265

[108] Oliphant CJ Hwang YY Walker JA Salimi M Wong SH Brewer JM. MHCII-mediated dialog between group 2 innate lymphoid cells and CD4+ T cells potentiates type 2 immunity and promotes parasitic helminth expulsion. Immunity. (2014) 41:283–95. 10.1016/j.immuni.2014.06.016 25088770 PMC4148706

[109] Halim TYF Steer CA Mathä L Gold MJ Martinez-Gonzalez I McNagny KM. Group 2 innate lymphoid cells are critical for the initiation of adaptive T helper 2 cell-mediated allergic lung inflammation. Immunity. (2014) 40:425–35. 10.1016/j.immuni.2014.01.011 24613091 PMC4210641

[110] Turner JE Stockinger B Helmby H. IL-22 mediates goblet cell hyperplasia and worm expulsion in intestinal helminth infection. PLoS Pathog. (2013) 9:e1003698. 10.1371/journal.ppat.1003698 24130494 PMC3795034

[111] Broadhurst MJ Leung JM Kashyap V McCune JM Mahadevan U McKerrow JH. IL-22+ CD4+ T cells are associated with therapeutic *Trichuris trichiura* infection in an ulcerative colitis patient. Sci Transl Med. (2010) 2:60ra88. 10.1126/scitranslmed.3001500 21123809

[112] Gaze S McSorley HJ Daveson J Jones D Bethony JM Oliveira LM. Characterising the mucosal and systemic immune responses to experimental human hookworm infection. PLoS Pathog. (2012) 8:e1002520. 10.1371/journal.ppat.1002520 22346753 PMC3276555

[113] Hasnain SZ Tauro S Das I Tong H Chen AH Jeffery PL. IL-10 promotes production of intestinal mucus by suppressing protein misfolding and endoplasmic reticulum stress in goblet cells. Gastroenterology. (2013) 144:357–68.e9. 10.1053/j.gastro.2012.10.043 23123183

[114] Hsu HP Lai MD Lee JC Yen MC Weng TY Chen WC. Mucin 2 silencing promotes colon cancer metastasis through interleukin-6 signaling. Sci Rep. (2017) 7:5823. 10.1038/s41598-017-04952-7 28725043 PMC5517441

[115] Shan YS Hsu HP Lai MD Yen MC Fang JH Weng TY. Suppression of mucin 2 promotes interleukin-6 secretion and tumor growth in an orthotopic immune-competent colon cancer animal model. Oncol Rep. (2014) 32:2335–42. 10.3892/or.2014.3544 25322805 PMC4240497

[116] Cohan VL Scott AL Dinarello CA Prendergast RA Interleukin-1 is a mucus secretagogue. Cell Immunol. (1991) 136:425–34. 10.1016/0008-8749(91)90364-H 1873825

[117] Sharba S Navabi N Padra M Persson JA Quintana-Hayashi MP Gustafsson JK. Interleukin 4 induces rapid mucin transport, increases mucus thickness and quality and decreases colitis and *Citrobacter rodentium* in contact with epithelial cells. Virulence. (2019) 10:97–117. 10.1080/21505594.2019.1573050 30665337 PMC6363059

[118] Yoo BB Mazmanian SK. The enteric network: interactions between the immune and nervous systems of the gut. Immunity. (2017) 46:910–26. 10.1016/j.immuni.2017.05.011 28636959 PMC5551410

[119] Satoh Y Ishikawa K Oomori Y Takeda S Ono K. Bethanechol and a G-protein activator, NaF/AlCl3, induce secretory response in Paneth cells of mouse intestine. Cell Tissue Res. (1992) 269:213–20. 10.1007/BF00319611 1358451

[120] Gustafsson JK Ermund A Ambort D Johansson MEV Nilsson HE Thorell K. Bicarbonate and functional CFTR channel are required for proper mucin secretion and link cystic fibrosis with its mucus phenotype. J Exp Med. (2012) 209:1263–72. 10.1084/jem.20120562 22711878 PMC3405509

[121] McLean LP Smith A Cheung L Sun R Grinchuk V Vanuytsel T. Type 3 muscarinic receptors contribute to clearance of *Citrobacter rodentium*. Inflamm Bowel Dis. (2015) 21:1860–71. 10.1097/MIB.0000000000000408 25985244 PMC4821008

[122] Lundgren O Jodal M Jansson M Ryberg AT Svensson L. Intestinal epithelial stem/progenitor cells are controlled by mucosal afferent nerves. PLoS ONE. (2011) 6:e16295. 10.1371/journal.pone.0016295 21347406 PMC3036584

[123] Kojima Y Ishihara K Komuro Y Saigenji K Hotta K Effects of the muscarinic receptor agonist carbachol and/or antagonist pirenzepine on gastric mucus secretion in rats. Scand J Gastroenterol. (1993) 28:647–51. 10.3109/00365529309096105 7689745

[124] Darby M Schnoeller C Vira A Culley F Bobat S Logan E. The M3 muscarinic receptor is required for optimal adaptive immunity to helminth and bacterial infection. PLoS Pathog. (2015) 11:e1004636. 10.1371/journal.ppat.1004636 25629518 PMC4309615

[125] McLean LP Smith A Cheung L Urban JF Sun R Grinchuk V. Type 3 muscarinic receptors contribute to intestinal mucosal homeostasis and clearance of nippostrongylus brasiliensis through induction of TH2 cytokines. Am J Physiol Gastrointest Liver Physiol. (2016) 311:G130–41. 10.1152/ajpgi.00461.2014 27173511 PMC4967171

[126] Schwerdtfeger LA Tobet SA. Vasoactive intestinal peptide regulates ileal goblet cell production in mice. Physiol Rep. (2020) 8:e14363. 10.14814/phy2.14363 32026594 PMC7002535

[127] Wu X Conlin VS Morampudi V Ryz NR Nasser Y Bhinder G. Vasoactive intestinal polypeptide promotes intestinal barrier homeostasis and protection against colitis in mice. PLoS ONE. (2015) 10:e0125225. 10.1371/journal.pone.0125225 25932952 PMC4416880

[128] Harmar A Arimura A Gozes I Journot L Laburthe M Pisegna JR. International union of pharmacology. XVIII. Nomenclature of receptors for vasoactive intestinal peptide and pituitary adenylate cyclase-activating polypeptide. Pharmacol Rev. (1998) 50:265–70. 9647867 PMC6721840

[129] Jayawardena D Guzman G Gill RK Alrefai WA Onyuksel H Dudeja PK. Expression and localization of VPAC1, the major receptor of vasoactive intestinal peptide along the length of the intestine. Am J Physiol Liver Physiol. (2017) 313:G16–25. 10.1152/ajpgi.00081.2017 28385693 PMC5538834

[130] Groneberg DA Hartmann P Dinh QT Fischer A. Expression and distribution of vasoactive intestinal polypeptide receptor VPAC2 mRNA in human airways. Lab Investig. (2001) 81:749–55. 10.1038/labinvest.3780283 11351046

[131] Rios J Zoukhri D Rawe IM Hodges RR Zieske JD Dartt DA. Immunolocalization of muscarinic and VIP receptor subtypes and their role in stimulating goblet cell secretion. Invest Ophthalmol Vis Sci. (1999) 40:1102–111. 10235543

[132] Fan H Wang A Wang Y Sun Y Han J Chen W. Innate lymphoid cells: regulators of gut barrier function and immune homeostasis. J Immunol Res. (2019) 2019:2525984. 10.1155/2019/2525984 31930146 PMC6942837

[133] Cardoso V Chesné J Ribeiro H Garcia-Cassani B Carvalho T Bouchery T. Neuronal regulation of type 2 innate lymphoid cells via neuromedin U. Nature. (2017) 549:277–81. 10.1038/nature23469 28869974 PMC5714273

[134] Wallrapp A Riesenfeld SJ Burkett PR Abdulnour REE Nyman J Dionne D. The neuropeptide NMU amplifies ILC2-driven allergic lung inflammation. Nature. (2017) 549:351–6. 10.1038/nature24029 28902842 PMC5746044

[135] Klose CS Artis D. Neuronal regulation of innate lymphoid cells. Curr Opin Immunol. (2019) 56:94–9. 10.1016/j.coi.2018.11.002 30530300

[136] Xavier RJ Podolsky DK. Unravelling the pathogenesis of inflammatory bowel disease. Nature. (2007) 448:427–34. 10.1038/nature06005 17653185

[137] Mak WY Zhao M Ng SC Burisch J. The epidemiology of inflammatory bowel disease: east meets west. J Gastroenterol Hepatol. (2019) 35:380–9. 10.1111/jgh.14872 31596960

[138] Sheng YH Hasnain SZ Florin THJ McGuckin MA. Mucins in inflammatory bowel diseases and colorectal cancer. J Gastroenterol Hepatol. (2012) 27:28–38. 10.1111/j.1440-1746.2011.06909.x 21913981

[139] Franke A McGovern DPB Barrett JC Wang K Radford-Smith GL Ahmad T. Genome-wide meta-analysis increases to 71 the number of confirmed Crohn's disease susceptibility loci. Nat Genet. (2010) 42:1118–25. 10.1038/ng.717 21102463 PMC3299551

[140] Barrett JC Hansoul S Nicolae DL Cho JH Duerr RH Rioux JD. Genome-wide association defines more than 30 distinct susceptibility loci for Crohn's disease. Nat Genet. (2008) 40:955–62. 10.1038/ng.175 18587394 PMC2574810

[141] Raouf AH Tsai HH Parker N Hoffman J Walker RJ Rhodes JM. Sulphation of colonic and rectal mucin in inflammatory bowel disease: reduced sulphation of rectal mucus in ulcerative colitis. Clin Sci. (1992) 83:623–6. 10.1042/cs0830623 1335401

[142] Johansson MEV Gustafsson JK Holmen-Larsson J Jabbar KS Xia L Xu H. Bacteria penetrate the normally impenetrable inner colon mucus layer in both murine colitis models and patients with ulcerative colitis. Gut. (2014) 63:281–91. 10.1136/gutjnl-2012-303207 23426893 PMC3740207

[143] Van Der Post S Jabbar KS Birchenough G Arike L Akhtar N Sjovall H. Structural weakening of the colonic mucus barrier is an early event in ulcerative colitis pathogenesis. Gut. (2019) 68:2142–51. 10.1136/gutjnl-2018-317571 30914450 PMC6872445

[144] Gersemann M Becker S Kübler I Koslowski M Wang G Herrlinger KR. Differences in goblet cell differentiation between Crohn's disease and ulcerative colitis. Differentiation. (2009) 77:84–94. 10.1016/j.diff.2008.09.008 19281767

[145] Strugala V Dettmar PW Pearson JP. Thickness and continuity of the adherent colonic mucus barrier in active and quiescent ulcerative colitis and Crohn's disease. Int J Clin Pract. (2008) 62:762–9. 10.1111/j.1742-1241.2007.01665.x 18194279

[146] Pullan RD Thomas GAO Rhodes M Newcombe RG Williams GT. Thickness of adherent mucus gel on colonic mucosa in humans and its relevance to colitis. Gut. (1994) 35:353–9. 10.1136/gut.35.3.353 8150346 PMC1374589

[147] Van Klinken BJW Van Der Wal JWG Einerhand A Büller HA Dekker J. Sulphation and secretion of the predominant secretory human colonic mucin MUC2 in ulcerative colitis. Gut. (1999) 44:387–93. 10.1136/gut.44.3.387 10026326 PMC1727426

[148] Forgue-Lafitte M-E Fabiani B Levy PP Maurin N Fléjou J-F Bara J. Abnormal expression of M1/MUC5AC mucin in distal colon of patients with diverticulitis, ulcerative colitis and cancer. Int J Cancer. (2007) 121:1543–9. 10.1002/ijc.22865 17565737

[149] Longman RJ Poulsom R Corfield AP Warren BF Wright NA Thomas MG. Alterations in the composition of the supramucosal defense barrier in relation to disease severity of ulcerative colitis. J Histochem Cytochem. (2006) 54:1335–48. 10.1369/jhc.5A6904.2006 16924127 PMC3958115

[150] Yamamoto-Furusho JK Mendivil-Rangel EJ Fonseca-Camarillo G. Reduced expression of mucin 9 (MUC9) in patients with ulcerative colitis. Inflamm Bowel Dis. (2012) 18:E601. 10.1002/ibd.21920 22028245

[151] Yamamoto-Furusho JK Ascaño-Gutiérrez I Furuzawa-Carballeda J Fonseca-Camarillo G. Differential expression of MUC12, MUC16, and MUC20 in patients with active and remission ulcerative colitis. Mediat Inflamm. (2015) 2015:659018. 10.1155/2015/659018 26770020 PMC4684874

[152] Parker N Tsai HH Ryder SD Raouf AH Rhodes JM. Increased rate of sialylation of colonic mucin by cultured ulcerative colitis mucosal explants. Digestion. (1995) 56:52–56. 10.1159/000201222 7895933

[153] Larsson JMH Karlsson H Crespo JG Johansson MEV Eklund L Sjövall H. Altered O-glycosylation profile of MUC2 mucin occurs in active ulcerative colitis and is associated with increased inflammation. Inflamm Bowel Dis. (2011) 17:2299–307. 10.1002/ibd.21625 21290483

[154] Niv Y. Mucin gene expression in the intestine of ulcerative colitis patients: a systematic review and meta-analysis. Eur J Gastroenterol Hepatol. (2016) 25:351–7. 10.1097/MEG.0000000000000707 27442499

[155] Buisine MP Desreumaux P Leteurtre E Copin MC Colombel JF Porchet N. Mucin gene expression in intestinal epithelial cells in Crohn's disease. Gut. (2001) 49:544–51. 10.1136/gut.49.4.544 11559653 PMC1728475

[156] Buisine MP Desreumaux P Debailleul V Gambiez L Geboes K Ectors N. Abnormalities in mucin gene expression in Crohn's disease. Inflamm Bowel Dis. (1999) 5:24–32. 10.1097/00054725-199902000-00004 10028446

[157] Bray F Ferlay J Soerjomataram I Siegel RL Torre LA Jemal A. Global cancer statistics 2018: GLOBOCAN estimates of incidence and mortality worldwide for 36 cancers in 185 countries. CA Cancer J Clin. (2018) 68:394–424. 10.3322/caac.21492 30207593

[158] Nakamori S Ota DM Cleary KR Shirotani K Irimura T. MUC1 mucin expression as a marker of progression and metastasis of human colorectal carcinoma. Gastroenterology. (1994) 106:353–61. 10.1016/0016-5085(94)90592-4 7905449

[159] Cao Y Blohm D Ghadimi BM Stosiek P Xing PX Karsten U. Mucins (MUC1 and MUC3) of gastrointestinal and breast epithelia reveal different and heterogeneous tumor-associated aberrations in glycosylation. J Histochem Cytochem. (1997) 45:1547–57. 10.1177/002215549704501111 9358856

[160] Ajioka Y Allison LJ Jass JR. Significance of MUC 1 and MUC2 mucin expression in colorectal cancer. JClin Pathol. (1996) 49:560–4. 10.1136/jcp.49.7.560 8813954 PMC500570

[161] Chang SK Dohrman AF Basbaum CB Ho SB Tsuda T Toribara NW. Localization of mucin (MUC2 and MUC3) messenger RNA and peptide expression in human normal intestine and colon cancer. Gastroenterology. (1994) 107:28–36. 10.1016/0016-5085(94)90057-4 8020672

[162] Byrd JC Bresalier RS. Mucins and mucin binding proteins in colorectal cancer. Cancer Metastasis Rev. (2004) 23:77–99. 10.1023/A:1025815113599 15000151

[163] De Rosa M Pace U Rega D Costabile V Duraturo F Izzo P. Genetics, diagnosis and management of colorectal cancer (review). Oncol Rep. (2015) 34:1087–96. 10.3892/or.2015.4108 26151224 PMC4530899

[164] Okayasu I Hatakeyama S Yamada M Ohkusa T Inagaki Y Nakaya R. A novel method in the induction of reliable experimental acute and chronic ulcerative colitis in mice. Gastroenterology. (1990) 98:694–702. 10.5555/URI:PII:001650859090290H 1688816

[165] Egger B Bajaj-Elliott M MacDonald TT Inglin R Eysselein VE Büchler MW. Characterisation of acute murine dextran sodium sulphate colitis: cytokine profile and dose dependency. Digestion. (2000) 62:240–8. 10.1159/000007822 11070407

[166] Renes IB Boshuizen JA Van Nispen DJPM Bulsing NP Büller HA Dekker J. Alterations in Muc2 biosynthesis and secretion during dextran sulfate sodium-induced colitis. Am J Physiol Gastrointest Liver Physiol. (2002) 282:G382–9. 10.1152/ajpgi.00229.2001 11804861

[167] Dharmani P Leung P Chadee K. Tumor necrosis factor-α and Muc2 mucin play major roles in disease onset and progression in dextran sodium sulphate-induced colitis. PLoS ONE. (2011) 6:e25058. 10.1371/journal.pone.0025058 21949848 PMC3176316

[168] Johansson ME V Gustafsson JK Sjöberg KE Petersson J Holm L Sjövall H. Bacteria penetrate the inner mucus layer before inflammation in the dextran sulfate colitis model. PLoS ONE. (2010) 5:e12238. 10.1371/journal.pone.0012238 20805871 PMC2923597

[169] Van der Sluis M De Koning BAE De Bruijn ACJM Velcich A Meijerink JPP Van Goudoever JB. Muc2-deficient mice spontaneously develop colitis, indicating that MUC2 is critical for colonic protection. Gastroenterology. (2006) 131:117–29. 10.1053/j.gastro.2006.04.020 16831596

[170] Das S Rachagani S Sheinin Y Smith LM Gurumurthy CB Roy HK. Mice deficient in Muc4 are resistant to experimental colitis and colitis-associated colorectal cancer. Oncogene. (2016) 35:2645–54. 10.1038/onc.2015.327 26364605 PMC5555307

[171] Petersson J Schreiber O Hansson GC Gendler SJ Velcich A Lundberg JO. Importance and regulation of the colonic mucus barrier in a mouse model of colitis. Am J Physiol Liver Physiol. (2011) 300:G327–33. 10.1152/ajpgi.00422.2010 21109593 PMC3302190

[172] Malmberg EK Noaksson KA Phillipson M Johansson ME V Hinojosa-Kurtzberg M Holm L. Increased levels of mucins in the cystic fibrosis mouse small intestine, and modulator effects of the Muc1 mucin expression. Am J Physiol Gastrointest Liver Physiol. (2006) 291:G203–10. 10.1152/ajpgi.00491.2005 16500918

[173] Scheinin T Butler DM Salway F Scallon B Feldmann M. Validation of the interleukin-10 knockout mouse model of colitis: antitumour necrosis factor-antibodies suppress the progression of colitis. Clin Exp Immunol. (2003) 133:38–43. 10.1046/j.1365-2249.2003.02193.x 12823276 PMC1808754

[174] Dosh RH Jordan-Mahy N Sammon C Maitre C Le. Interleukin 1 is a key driver of inflammatory bowel disease-demonstration in a murine IL-1Ra knockout model. Oncotarget. (2019) 10:3559. 10.18632/oncotarget.26894 31191826 PMC6544399

[175] Stremmel W Staffer S Schenieder MJ Gan-Schreier H Wannhoff A Stuhrmann N. Genetic mouse models with intestinal-specific tight junction deletion resemble an ulcerative colitis phenotype. J Crohns Colitis. (2017) 11:1247–57. 10.1093/ecco-jcc/jjx075 28575164 PMC5881657

[176] Bento CF Renna M Ghislat G Puri C Ashkenazi A Vicinanza M. Mammalian autophagy: how does it work? Annu Rev Biochem. (2016) 85:685–713. 10.1146/annurev-biochem-060815-014556 26865532

[177] Haq S Grondin J Banskota S Khan WI. Autophagy: roles in intestinal mucosal homeostasis and inflammation. J Biomed Sci. (2019) 26:19. 10.1186/s12929-019-0512-2 30764829 PMC6375151

[178] Hampe J Franke A Rosenstiel P Till A Teuber M Huse K. A genome-wide association scan of nonsynonymous SNPs identifies a susceptibility variant for Crohn disease in ATG16L1. Nat Genet. (2007) 39:207–11. 10.1038/ng1954 17200669

[179] Tsuboi K Nishitani M Takakura A Imai Y Komatsu M Kawashima H. Autophagy protects against colitis by the maintenance of normal gut microflora and secretion of mucus. J Biol Chem. (2015) 290:20511–26. 10.1074/jbc.M114.632257 26149685 PMC4536456

[180] Fu J Wei B Wen T Johansson MEV Liu X Bradford E. Loss of intestinal core 1-derived O-glycans causes spontaneous colitis in mice. J Clin Invest. (2011) 121:1657–66. 10.1172/JCI45538 21383503 PMC3069788

[181] Bergstrom K Fu J Johansson MEV Liu X Gao N. Core 1- and 3-derived O-glycans collectively maintain the colonic mucus barrier and protect against spontaneous colitis in mice. Mucosal Immunol. (2017) 10:91–103. 10.1038/mi.2016.45 27143302 PMC5097036

[182] Kawashima H. Roles of the gel-forming MUC2 mucin and its O-glycosylation in the protection against colitis and colorectal cancer. Biol Pharm Bull. (2012) 35:1637–41. 10.1248/bpb.b12-00412 23037153

[183] Bergstrom K Liu X Zhao Y Gao N Wu Q Song K. Defective intestinal mucin-type O-glycosylation causes spontaneous colitis-associated cancer in mice. Gastroenterology. (2016) 151:152–64.e11. 10.1053/j.gastro.2016.03.039 27059389 PMC5068133

[184] An G Wei B Xia B McDaniel JM Ju T Cummings RD. Increased susceptibility to colitis and colorectal tumors in mice lacking core 3-derived O-glycans. J Exp Med. (2007) 204:1417–29. 10.1084/jem.20061929 17517967 PMC2118614

[185] Vos T Allen C Arora M Barber RM Brown A Carter A Global, regional, and national incidence, prevalence, and years lived with disability for 310 diseases and injuries, 1990–2015: a systematic analysis for the Global Burden of Disease Study 2015. Lancet. (2016) 388:1545–602. 10.1016/S0140-6736(16)31678-6 27733282 PMC5055577

[186] Klementowicz JE Travis MA Grencis RK. *Trichuris muris*: a model of gastrointestinal parasite infection. Semin Immunopathol. (2012) 34:815–28. 10.1007/s00281-012-0348-2 23053395 PMC3496546

[187] Hasnain SZ Wang H Ghia J-E Haq N Deng Y Velcich A. Mucin gene deficiency in mice impairs host resistance to an enteric parasitic infection. Gastroenterology. (2010) 138:1763–71. 10.1053/j.gastro.2010.01.045 20138044 PMC3466424

[188] Hasnain SZ McGuckin MA Grencis RK Thornton DJ. Serine protease(s) secreted by the nematode *Trichuris muris* degrade the mucus barrier. PLoS Negl Trop Dis. (2012) 6:e1856. 10.1371/journal.pntd.0001856 23071854 PMC3469553

[189] Leon-Coria A Kumar M Chadee K. The delicate balance between *Entamoeba histolytica*, mucus and microbiota. Gut Microbes. (2020) 11:118–25. 10.1080/19490976.2019.1614363 31091163 PMC6973333

[190] Chadee K Petri WA Innes DJ Ravdin JI. Rat and human colonic mucins bind to and inhibit adherence lectin of *Entamoeba histolytica*. J Clin Invest. (1987) 80:1245–54. 10.1172/JCI113199 2890655 PMC442377

[191] Lidell ME Moncada DM Chadee K Hansson GC. Entamoeba histolytica cysteine protease cleave the MUC2 mucin in its C-terminal domain and dissolve the protective colonic mucus gel. Proc Natl Acad Sci USA. (2006) 103:9298–303. 10.1073/pnas.0600623103 16754877 PMC1482604

[192] Thibeaux R Weber C Hon C-C Dillies M-A Avé P Coppée J-Y. Identification of the virulence landscape essential for *Entamoeba histolytica* invasion of the human colon. PLoS Pathog. (2013) 9:e1003824. 10.1371/journal.ppat.1003824 24385905 PMC3868522

[193] Cornick S Moreau F Chadee K. Entamoeba histolytica cysteine proteinase 5 evokes mucin exocytosis from colonic goblet cells via αvβ3 integrin. PLoS Pathog. (2016) 12:e1005579. 10.1371/journal.ppat.1005579 27073869 PMC4830554

[194] Leon-Coria A Kumar M Moreau F Chadee K. Defining cooperative roles for colonic microbiota and Muc2 mucin in mediating innate host defense against *Entamoeba histolytica*. PLoS Pathog. (2018) 14:e1007466. 10.1371/journal.ppat.1007466 30500860 PMC6268003

[195] Cobo ER Kissoon-Singh V Moreau F Holani R Chadee K. MUC2 mucin and butyrate contribute to the synthesis of the antimicrobial peptide cathelicidin in response to *E. histolytica* and DSS-induced colitis. Infect Immun. (2017) 85:e00905-16. 10.1128/IAI.00905-16 28069814 PMC5328487

[196] He M Miyajima F Roberts P Ellison L Pickard DJ Martin MJ. Emergence and global spread of epidemic healthcare-associated *Clostridium difficile*. Nat Genet. (2013) 45:109–13. 10.1038/ng.2478 23222960 PMC3605770

[197] Chen X Katchar K Goldsmith JD Nanthakumar N Cheknis A Gerding DN. A mouse model of *Clostridium difficile*-associated disease. Gastroenterology. (2008) 135:1984–92. 10.1053/j.gastro.2008.09.002 18848941

[198] Engevik MA Yacyshyn MB Engevik KA Wang J Darien B Hassett DJ. Human *Clostridium difficile* infection: altered mucus production and composition. Am J Physiol Liver Physiol. (2015) 308:G510–24. 10.1152/ajpgi.00091.2014 25552581 PMC4422372

[199] Frisbee AL Saleh MM Young MK Leslie JL Simpson ME Abhyankar MM. IL-33 drives group 2 innate lymphoid cell-mediated protection during *Clostridium difficile* infection. Nat Commun. (2019) 10:2712. 10.1038/s41467-019-10733-9 31221971 PMC6586630

[200] McAuley JL Linden SK Png CW King RM Pennington HL Gendler SJ. MUC1 cell surface mucin is a critical element of the mucosal barrier to infection. J Clin Invest. (2007) 117:2313–24. 10.1172/JCI26705 17641781 PMC1913485

[201] Wang L Llorente C Hartmann P Yang AM Chen P Schnabl B. Methods to determine intestinal permeability and bacterial translocation during liver disease. J Immunol Methods. (2015) 421:44–53. 10.1016/j.jim.2014.12.015 25595554 PMC4451427

[202] Zarepour M Bhullar K Montero M Ma C Huang T Velcich A. The mucin Muc2 limits pathogen burdens and epithelial barrier dysfunction during *Salmonella enterica* serovar *Typhimurium colitis*. Infect Immun. (2013) 81:3672–83. 10.1128/IAI.00854-13 23876803 PMC3811786

[203] Chakravarty B Gu Z Chirala SS Wakil SJ Quiocho FA. Human fatty acid synthase: structure and substrate selectivity of the thioesterase domain. Proc Natl Acad Sci USA. (2004) 101:15567–72. 10.1073/pnas.0406901101 15507492 PMC524853

[204] Wei X Yang Z Rey FE Ridaura VK Davidson NO Gordon JI. Fatty acid synthase modulates intestinal barrier function through palmitoylation of mucin 2. Cell Host Microbe. (2012) 11:140–52. 10.1016/j.chom.2011.12.006 22341463 PMC3285413

[205] Dawson PA Huxley S Gardiner B Tran T McAuley JL Grimmond S. Reduced mucin sulfonation and impaired intestinal barrier function in the hyposulfataemic NaS1 null mouse. Gut. (2009) 58:910–9. 10.1136/gut.2007.147595 19201772

[206] Maroni LJ van de Graaf SF Hohenester SDJ Oude Elferink RP Beuers U. Fucosyltransferase 2: a genetic risk factor for primary sclerosing cholangitis and Crohn's disease-a comprehensive review. Clinic Rev Allerg Immunol. (2015) 48:182–91. 10.1007/s12016-014-8423-1 24828903

[207] Magalhães A Rossez Y Robbe-Masselot C Maes E Gomes J Shevtsova A. Muc5ac gastric mucin glycosylation is shaped by FUT2 activity and functionally impacts *Helicobacter pylori* binding. Sci Rep. (2016) 6:25575. 10.1038/srep25575 27161092 PMC4861914

[208] Schroten H Hanisch F-G Hansman GS Human norovirus interactions with histo-blood group antigens and human milk oligosaccharides. J Virol. (2016) 90:5855–9. 10.1128/jvi.00317-16 27122582 PMC4907220

[209] Hang HC Bertozzi CR. The chemistry and biology of mucin-type O-linked glycosylation. Bioorganic Med Chem. (2005) 13:5021–34. 10.1016/j.bmc.2005.04.085 16005634

[210] Hasnain SZ Dawson PA Lourie R Hutson P Tong H Grencis RK. Immune-driven alterations in mucin sulphation is an important mediator of *Trichuris muris* helminth expulsion. PLoS Pathog. (2017) 13:e1006218. 10.1371/journal.ppat.1006218 28192541 PMC5325613

